# Dopamine receptor D3 is related to prognosis in human hepatocellular carcinoma and inhibits tumor growth

**DOI:** 10.1186/s12885-022-10368-y

**Published:** 2022-12-02

**Authors:** Yan Yan, Yonghua Chen, Jiahao Pan, Wei Xing, Qiang Li, Yan Wang, Liba Gei, Yunfei Yuan, Jingdun Xie, Weian Zeng, Dongtai Chen

**Affiliations:** 1 Department of Anesthesiology, Huizhou Municipal Central Hospital, Huizhou, 516001 China; 2 Department of Anesthesiology, State Key Laboratory of Oncology in South China, Collaborative Innovation Center for Cancer Medicine, Sun Yat-Sen University Cancer Center, Guangzhou, 510060 China; 3 Department of Anesthesiology, Peking University Shenzhen Hospital, Shenzhen, 518000 China; 4 Department of Anesthesiology, Inner Mongolia Autonomous Region Cancer Hospital/Affiliated People’s Hospital of Inner Mongolia Medical University, Hohhot, 010010 China; 5 Department of Hepatobiliary Oncology, State Key Laboratory of Oncology in South China, Collaborative Innovation Center for Cancer Medicine, Sun Yat-Sen University Cancer Center, Guangzhou, 510060 China

**Keywords:** Dopamine receptor D3, Hepatocellular carcinoma, Prognosis, Target therapy, ERK pathway

## Abstract

**Background:**

Dopamine receptors have been reported to play important roles in cancer progression. However, the role of dopamine receptor D3 (DRD3) in hepatocellular carcinoma (HCC) remains unclear.

**Methods:**

The expression of DRD3 was detected by immunohistochemistry and real-time qPCR. The prognostic value of DRD3 in patients was investigated by analyzing selected databases, including cBioPortal and Kaplan–Meier plotter. Cell growth was tested by CCK8 assay, and Transwell assays were performed to assess cancer cell migration and invasion. The cAMP/ERK/CREB signaling pathway was evaluated by Western blot analysis and ELISA. An HCC xenograft model was established for in vivo experiments.

**Results:**

DRD3 mRNA expression was significantly higher in nontumor tissues than in tumor tissues. Lower protein expression of DRD3 was related to poor recurrence-free survival (RFS) and overall survival (OS). Kaplan–Meier plotter analysis showed that higher expression of DRD3 mRNA was associated with better OS, RFS, disease-specific survival (DSS), and progression-free survival (PFS). cBioPortal analysis revealed that the alteration group, which harbored genetic mutations in DRD3, exhibited poor OS, RFS, DSS and PFS. According to CCK8 and Transwell assays, stable DRD3 overexpression cell line (ex-DRD3-SK-HEP-1) showed weaker proliferation, migration and invasion behaviors. PD128907, a DRD3 agonist, suppressed proliferation, migration and invasion in HCC cell lines, while U99194, a DRD3 antagonist, enhanced proliferation, migration and invasion in HCC cell lines. Western blot analysis and ELISA revealed that stable DRD3 knock-down cell line (sh-DRD3-PLC/PRF/5) and U99194 both increased the protein levels of cAMP, p-ERK and p-CREB; on the other hand, ex-DRD3-SK-HEP-1 and PD128907 decreased the protein levels of cAMP, p-ERK and p-CREB. SCH772984, an ERK antagonist, abolished the effect of U99194 on the malignant biological behaviors of HCC cells. In vivo, PD128907 suppressed tumor growth, and U99194 enhanced tumor growth*.*

**Conclusion:**

Our results suggest that down-regulation of DRD3 is strongly involved in the progression of HCC, and DRD3 might be consider as an independent prognostic factor for HCC. Furthermore, DRD3 agonists may be a promising strategy for HCC therapy.

**Supplementary Information:**

The online version contains supplementary material available at 10.1186/s12885-022-10368-y.

## Introduction

Liver cancer is one of the most common malignancies worldwide; it is the fourth leading cause of cancer-related death and the second most lethal cancer, causing an estimated 800,000 deaths each year. Among adults, hepatocellular carcinoma (HCC) accounts for 70–85% of liver malignancies [1–4]. The treatment of liver cancer is still based on radical resection. However, most patients are already in an advanced stage when the disease is found and lose the opportunity for surgery. This limitation leads to poor prognosis and therapeutic efficacy in patients with HCC [5]. The main nonsurgical treatment is chemotherapy. However, in recent years, with the discovery of various tumor target sites and the development of targeted drugs, targeted therapy has received increasing attention [6, 7].

Dopamine receptors are divided into D1-like (DRD1, DRD5) and D2-like (DRD2, DRD3, DRD4) receptors according to their different physicochemical properties [8, 9]. In recent years, there have been increasing numbers of reports on the effects of dopamine receptors on the occurrence and development of different types of tumors. Breast cancer and HCC patients positive for DRD1 expression have a poor prognosis [10, 11], and dopamine receptor inhibitors can destroy tumor cells by terminating the self-renewal of tumor stem cells [12]; moreover, they can also inhibit AKT signaling pathways, leading to suppression of breast tumor growth [13]. Furthermore, DRD2 expression in pancreatic cancer patients is increased, and DRD2-related blockers can inhibit activation of the ERK signaling pathway, thereby limiting tumor progression [14]. In these studies, the most commonly studied receptors were DRD1 and DRD2, while fewer studies have focused on the other receptors, especially DRD3. DRD3, a member of the D2-like receptor family, can suppress the activation of cAMP to cause diverse physiological and pathological effects, similar to DRD2. In the only studies in cancer, the expression of both DRD2 and DRD3 was found to be increased in breast cancer patients [15], and DRD2 and DRD3 were found to have the same expression levels in the cholangiocarcinoma cell lines Mz-chA-1 and SG231 [16]. These results suggested that DRD3 might have the same effect on tumors as DRD2.

It has been reported that DRD2 agonists can effectively inhibit the proliferation and metastasis of HCC cells [17], although there are still no reports on the effects of DRD3 on liver cancer, including the prognosis and biological behavior of HCC. This study aims to explore whether DRD3 can be used to predict the prognosis of HCC patients and to evaluate its effect on liver cancer cells in order to preliminarily reveal the role of DRD3 in the occurrence and development of liver cancer and lay a foundation for further research in the future.

## Material and methods

### HCC patients and tissue specimens

The Clinical Research Ethics Committee of Sun Yat-sen University Cancer Center (Guangzhou, China) had approved this project, and we had got all the informed consents from the subjects. All data and material were obtained in accordance with the Declaration of Helsinki. In this study, tumor tissue samples from 218 HCC patients were used for immunohistochemical staining, and liver cancer tissues and adjacent non-liver cancer tissues from another 74 patients were collected for real-time qPCR. All patients underwent resection of hepatocellular carcinoma performed by the same surgeon between 2003 and 2008 at the Department of Hepatobiliary Oncology, Sun Yat-sen University Cancer Center (Guangzhou, China). There were 187 males and 31 females, with an average age of 48.7 years (range: 20–79 years). The median follow-up period was 54 months (range: 1–127 months).

### Cell lines and reagents

All the cell lines and reagents were prepared as previously described[10]. HCC cell lines including MHCC97-H, MHCC97-L, SK-HEP-1, PLC/PRF/5, and Huh-7, and a normal liver cell line (Miha) were purchased from the Chinese Type Culture Collection (Shanghai, China), while Hep-G2 and Hep-3B cells were purchased from Guangzhou Cellcook Biotech Co., Ltd. (Guangzhou, China). Cells were cultured with Dulbecco’s modified Eagle’s medium (DMEM, Gibco, Carlsbad, CA, USA) supplemented with 100 μg/mL penicillin, 100 μg/mL streptomycin (Invitrogen, Carlsbad, CA, USA) and 10% fetal bovine serum (Gibco, USA) in a 37 °C incubator with 5% carbon dioxide and 85% humidity. Cells were cultured for three to six passages.

The sequences used for overexpression and shRNA-mediated knockdown of DRD3 were designed by GeneCopoeia (Rockville, MD, USA), and the pEZ-Lv105 human DRD3 overexpression vector, psi-LVRU6GP human DRD3 shRNA vector and pEZ-Lv105-GFP control vector were constructed by GeneCopoeia (Rockville, MD, USA). Base on the results of examining DRD3 expression in HCC cell lines, PLC/PRF/5 was chosen for constructing stable DRD3 knock-down cell lines while SK-HEP-1 was chosen for constructing stable DRD3 overexpression cell line. HCC cells needed to be 70% confluent before transfection was performed. Lenti-Pac™ HIV Expression Packaging Kits (GeneCopoeia, Atlanta, GA, USA) were used before transfection according to the manufacturer’s instructions. After stably transfecting the cell lines, all the cell lines had been treated with puromycin (2–4 μg/ml) for at least two weeks. Stable DRD3 knock-down cell lines (sh-DRD3-PLC/PRF/5–1, sh-DRD3-PLC/PRF/5–2), control cell line (sh-NC-PLC/PRF/5), a stable DRD3 overexpression cell line (ex-DRD3-SK-HEP-1) and control cell line (ex-vector-SK-HEP-1) were constructed and Western blotting was used to verify whether the cell lines were established successfully.

Dimethyl sulfoxide (DMSO, 20–139), U99194 (U116) and PD128907 (P216) were purchased from Sigma (St. Louis, MO, USA). SCH772984 (S7101) was purchased from Selleck (Houston, Texas, USA). The anti-DRD3 antibody (LS-B13859) was purchased from Lifespan Biosciences (Seattle, WA, USA). Primary antibodies specific for ERK (4695S), p-ERK (4370S), CREB (9197S), p-CREB (9198S), and β-tubulin (2146S) and an anti-rabbit antibody (7074S), which served as a secondary antibody, were purchased from Cell Signaling Technology (Danvers, MA, USA).

### shinyGEO

shinyGEO (https://gdancik.shinyapps.io/shinyGEO/) is an online tool that can download gene expression data sets directly from The Gene Expression Omnibus (GEO) then analyze gene expression and perform survival analysis [18]. The cohort of GSE14520 [19] had been download and DRD3 expression was analyzed using GraphPad Software 6.

### Kaplan–meier plotter analysis

Kaplan–Meier plotter (kmplot.com/analysis/) is an online tool providing easy access to information about the associations of mRNA expression with the survival of patients with liver cancer, breast cancer, ovarian cancer, lung cancer and gastric cancer [20]. Based on the median values of mRNA expression, patients were divided into the high and low expression groups, and survival analysis was performed by the K-M method to generate survival curves. K-M plotter can show many results, including the number at risk (cases), HR, 95% CI and p value. Differences were considered statistically significant when *P* < 0.05. In this study, we evaluated the relationships between DRD3 and overall survival (OS), recurrence-free survival (RFS), disease-specific survival (DSS), and progression-free survival (PFS).

### cBioPortal

cBioPortal (www.cbioportal.org) is an online open access website resource for exploring, visualizing, and analyzing multidimensional cancer genomics data [21]. We used this online tool to analyze the associations between genetic mutations in DRD3 and OS, RFS, DSS and PFS. The survival curves are displayed on the webpage, and a difference was considered statistically significant when *P* < 0.05.

### Real-time qPCR

RNA was extracted from HCC tissues and cell lines using TRIzol (Invitrogen, Carlsbad, CA) according to the instructions. cDNA was obtained by reverse transcription with a PrimeScript RT Kit (Takara, Dalian, China). Primer pairs specific for DRD3 (Invitrogen) were used, and GAPDH (Supplementary Table 1) served as the endogenous control. SYBR Green qPCR Super Mix and an ABI7900HT sequence detection system were used to run the thermal cycling program, and the presence of only one peak per reaction curve was considered to indicate a valid result.

### Immunohistochemistry

Immunohistochemistry was performed as previously described [22]. Tissue samples from HCC patients were fixed with 100 mL/L formalin and were then embedded in paraffin for continuous sectioning. After the tissue slides were baked and dewaxed at 60 °C for 2 h, they were hydrated through an alcohol gradient and blocked with 30 mL/L H_2_O_2_ for 45 min. Antigen retrieval was conducted in citric acid buffer (pH 6.0) in a microwave at medium and high heat for 5 min each and medium heat for 15 min and were then incubated with the anti-DRD3 antibody (1:200) at 4 °C overnight. After repeated washes with PBS, the tissue slides were incubated with the secondary antibody in a 37 °C incubator for 30 min. A DAB reagent set (DAKO, Carpinteria, CA) was used to develop color, and nuclei were stained with hematoxylin. The results were assessed by two formally trained pathologists using a double-blind method with the German immune response score (IRS). Based on the staining intensity, DRD3 protein staining was scored as 0 = not stained, 1 = weakly stained, 2 = moderately stained, or 3 = strongly stained. In addition, the percentage of positive staining in tumor tissue was scored as 0 (< 10%), 1 (10%–25%), 2 (26%–50%), 3 (51%–75%) or 4 (> 75%). The two scores were multiplied and weighted for each case, and the cases were divided into four groups: absent staining (-), weak staining ( +), moderate staining (+ +), and strong staining (+ + +).

### KEGG

KEGG (https://www.kegg.jp/) is an online tool including eighteen databases categorized into systems, genomic, chemical and health information [23]. The possible signal pathways which might be regulated by DRD3 were predicted by KEGG mapping tools, and the copyright permission of KEGG had been obtained.

### Western blot analysis

Western blot analysis was performed as previously described [24]. Total protein of cells was extracted and separated by SDS–PAGE, then transferred to polyvinylidene fluoride (PVDF) membranes (Millipore, MA, USA). Base on the molecular weights of the proteins which should be examined, the membranes had been cut before the incubation with primary antibodies. According to the expected molecular weight of the target protein, the membranes had been cut for suitable size to incubate with primary antibodies (included 35–45 kDa for DRD3, included 45–60 kDa for β-tubulin, included 35–45 kDa for ERK and p-ERK, included 35–45 kDa for CREB and p-CREB). All the primary antibodies had been made the specific detection and the results had been uploaded as supplementary file. After overnight incubating with primary antibodies at 4 °C, Membranes were incubated with anti-rabbit secondary antibodiesat room temperature for 1 h. BeyoECL Plus (Beyotime Biotechnology, Shanghai, China) was used for visualization and imaging. The images of original blots are provided in the Supplementary Materials.

### Enzyme-linked immunosorbent assay (ELISA)

ELISA were performed by using ELISA Kits (Cat: LS-F10530, Lifespan Biosciences) which was used for examining the cAMP levels in cell lines and tumor tissues. All experimental steps were strictly carried out in accordance with the instructions.

### Cell viability

Cell viability was examined with the Cell Counting Kit 8 (CCK8, Dojindo, Shanghai, China) according to the previously described [25]. Cell lines were seeded in 96-well plates at a density of 6 × 10^3^ cells per well overnight and were then treated with PD128907, U99194, SCH772984 (1 µM) or 0.1% DMSO as the control for 24 h. CCK8 reagent was added to each well and incubated at 37 °C for 2 h. The absorbance of each well was detected at 450 nm (OD450). CCK8 assays of cells transfected with sh-NC-PLC/PRF/5, sh-DRD3-PLC/PRF/5, ex-vector-SK-HEP-1 and ex-DRD3-SK-HEP-1 were performed on Days 0, 1, 2, 3, 4 and 5. The remaining steps were exactly the same as those reported above.

### Colony formation assay

Colony formation assays were performed as the previously described [10]. About 1000 cells were seeded in each well. The colonies were washed by PBS after 14 days incubation. 4% paraformaldehyde was used to fixed the colonies for 30 min. The colonies were stained with 0.1% crystal violet, then counted the number of colonies and filmed the images.

### Wound healing assay

Wound healing assays were performed as the previously described [26]. Cells were planted into 12 well plates. Scratch the plates to make the wounds with a sterile pipette tip when the cells were up to 80%-90% confluence. After washing with PBS, DMEM without FBS was used to culture the cells, then the cell images were filmed.

### Cell migration and invasion assays

Transwell assays were used to examine cell migration and invasion and performed as the previously described [27]. Migration and invasion assays were performed according to the manufacturer’s instructions. 1.0 × 10^4^ cells were placed into top chamber of each insert (BD Biosciences) with PD128907 (100 nM), U99194 (1 nM), SCH772984 (1 µM) or 0.1% DMSO as the control. And the plates were incubated at 37 °C for 24 h. Sh-NC-PLC/PRF/5, sh-DRD3-PLC/PRF/5, ex-vector-SK-HEP-1 and ex-DRD3-SK-HEP-1 cells were examine using the same methods without drug treatment.

### Xenograft tumor formation

The in vivo experiments were performed as the previously described [10]. All protocols for animal experiments were approved by the Clinical Research Ethics Committee of Sun Yat-sen University Cancer Center under Project License L102012016002Q and conformed to relevant aspects of the ARRIVE guidelines [28].

Eighteen mice were randomly divided into the control group, U99194 group and PD128907 group, with 6 mice in each group. Xenografts were established in each mouse by subcutaneous injection of 1 × 10^6^ PLC/PRF/5 cells in 100 µl of PBS solution mixed with 100 µl of Matrigel. Approximately one week after inoculation, tumors were visible. Then, the mice were treated with PD128907 (0.1 mg/kg), U99194 (0.1 mg/kg), and 0.1% DMSO daily by intraperitoneal injection. Every 3rd day, tumor volumes were calculated with the formula a (length) × b^2^ (width)/2, and mice were weighed at the same time. After 30 days, the mice were sacrificed by isoflurane anesthesia followed by cervical dislocation, and the tumors were weighed post autopsy.

Eighteen mice were randomly divided into three groups. One group were injected with sh-NC-PLC/PRF/5 cells, while the other groups were injected with sh-DRD3-PLC/PRF/5 cells. One group with sh-DRD3-PLC/PRF/5 cells was treated with SCH772984 (2 mg/kg). Tumor volumes were calculated with the same formula. After 24 days, the mice were sacrificed by isoflurane anesthesia followed by cervical dislocation, and the tumors were prepared for other experiments.

### Statistical analysis

Statistical analysis was performed as the previously described [29, 30]. All data are presented as means with the respective standard errors of the mean (SEMs) and were analyzed using GraphPad Software 6 for Windows (GraphPad, La Jolla, CA, USA) and Statistical Package for the Social Sciences (SPSS, version 22.0). The chi-square test was used to verify the relationships between DRD3 and clinicopathological features. Kaplan–Meier survival analysis with the log-rank test was used to evaluate the relationships between DRD3 and overall survival or recurrence-free survival. A Cox proportional hazards regression model was used to perform multivariate analysis for all prognostic factors that were found to be significant in univariate analysis. Student's t test was used to analyze and compare differences between the two groups of data. One-way ANOVA was used to analyze data with homogeneity of variance from multiple groups, and the Bonferroni method was then used for pairwise comparisons when a statistically significant difference was identified by ANOVA. *P* < 0.05 was considered statistically significant. At least three replicates were performed for each experiment at the cellular level.

## Results

### DRD3 can be an independent prognostic factor for HCC survival

**Fig. 1:**
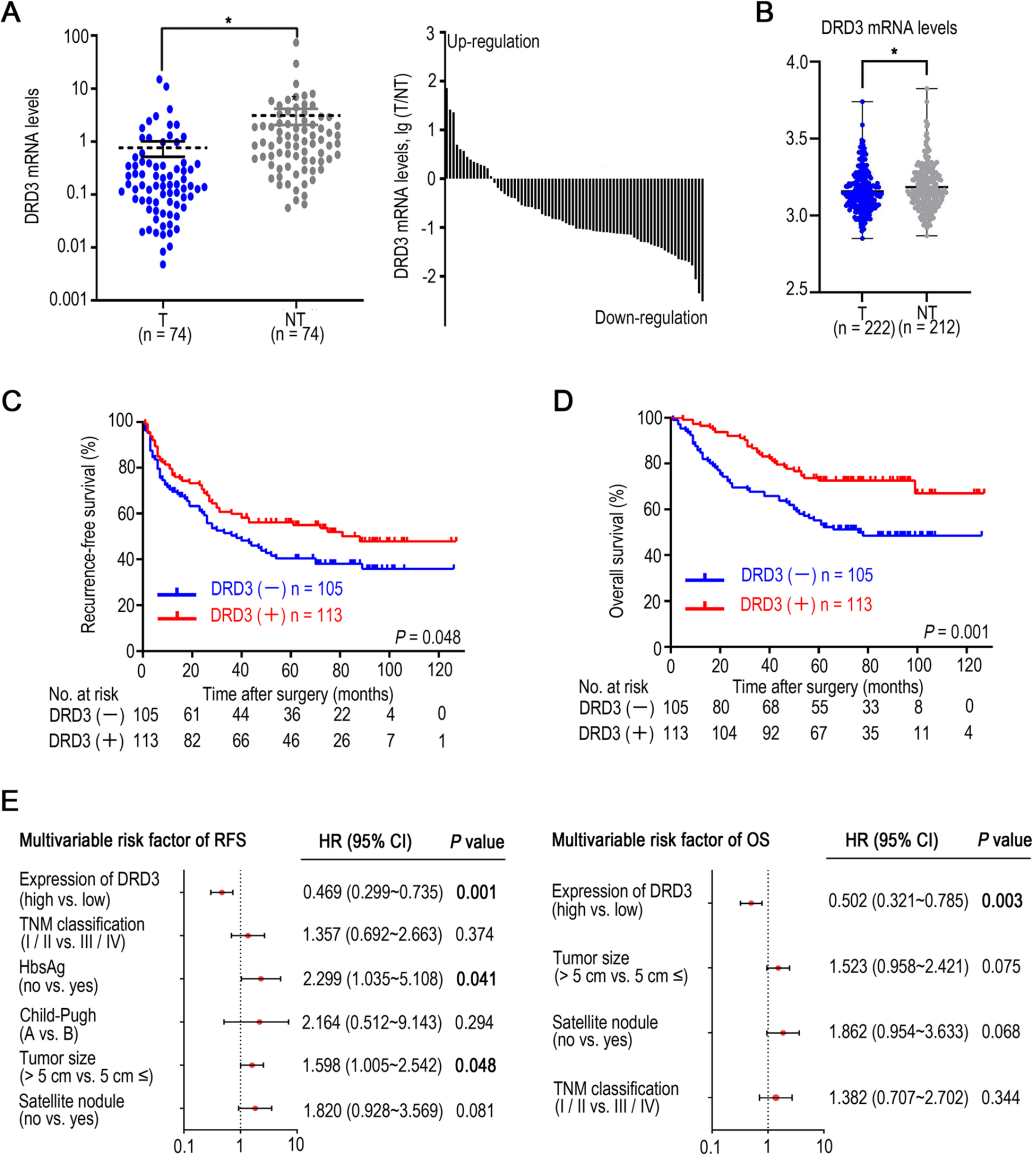
Expression of DRD3 in tumors and prognostic value of DRD3 in hepatocellular carcinoma (HCC). (**A**) DRD3 mRNA expression in 74 pairs of tumor tissues and adjacent nontumor tissues were detected by Real-Time qPCR. (**B**) DRD3 mRNA expression in tumor tissues and nontumor tissues base on the cohort of GSE14520. (**C**, **D**) Kaplan–Meier survival analysis was performed to assess the associations between DRD3 protein expression detected by IHC and recurrence-free survival (RFS) and overall survival (OS) in 218 HCC patients. (**E**) Multivariate Cox regression analyses of prognostic factors for RFS and OS in 218 HCC patients. * *P* < 0.05

First, DRD3 mRNA was detected in tumor and adjacent nontumor tissues. DRD3 mRNA expression was significantly higher in nontumor tissues than in tumor tissues (Fig. 1A), and the same result had been shown in the cohort of GSE14520 (Fig. 1B). Based on the Kaplan–Meier analysis results, lower DRD3 protein expression was related to poor recurrence-free survival (RFS, *P* = 0.048) and overall survival (OS, *P* = 0.001) (Fig. 1C and 1D). In addition, DRD3 expression was significantly associated with vascular invasion (*P* = 0.040) and TNM classification (*P* = 0.042) (Supplementary Table 2) according to the analysis of DRD3 and other clinicopathological characteristics. Based on the results of univariate analysis of all clinicopathological characteristics (Table 1), HBsAg status, Child–Pugh classification, tumor size, satellite nodule status, TNM classification and DRD3 expression were included in the multivariate Cox regression model. DRD3 was identified as a positive predictive factor for both RFS (*P* = 0.001, hazard ratio: 0.469, 95% CI: 0.299–0.735) and OS (*P* = 0.003, hazard ratio: 0.502, 95% CI: 0.321–0.785) (Fig. 1E and Table 1).

**Table 1:** Univariate and multivariate analyses of RFS and OS rates for 218 HCC patients after curative resection

RFS				OS		
Variables	Univariate*P*	Multivariable Analysis		Univariate*P*	Multivariable Analysis	
		HR (95% CI)	*P*		HR (95% CI)	*P*
Sex						
Female						
Male	0.789			0.816		
Age (y)						
≤ 50						
> 50	0.213			0.210		
HBsAg						
Negative						
Positive	**0.023**	2.299 (1.035–5.108)	**0.041**	0.137		
Child–Pugh classification^**l**^						
A						
B	**0.022**	2.164 (0.512–9.143)	0.294	0.230		
AFP (ng/ml)						
≤ 20						
20–400						
> 400	0.824			0.509		
GGT (units/L)						
≤ 50						
> 50	0.216			0.104		
Tumor size (cm)						
≤ 5						
> 5	**0.001**	1.598 (1.005–2.542)	**0.048**	**0.006**	1.523 (0.958–2.421)	0.075
Satellite nodules						
No						
Yes	**0.002**	1.820 (0.928–3.569)	0.081	**0.001**	1.862 (0.954–3.633)	0.068
Tumor capsule						
No						
Yes	0.196			0.688		
Vascular invasion						
No						
Yes	0.933			0.066		
Cirrhosis						
No						
Yes	0.308			0.988		
TNM classification^2^						
I/II						
III/IV	**0.037**	1.357 (0.692–2.663)	0.374	**0.001**	1.382 (0.707–2.702)	0.344
(Continued)						
DRD3 expression						
Negative						
Positive	**0.048**	0.469 (0.299–0.735)	**0.001**	**0.001**	0.502 (0.321–0.785)	**0.003**

^1^There was no patient with Child–Pugh Class C disease

^2^Tumor–node–metastasis (TNM) staging was evaluated based on the seventh edition of the American Joint Committee on Cancer (AJCC)

Abbreviations: DRD3, dopamine receptor D3. HCC, hepatocellular carcinoma. AFP, Alpha-fetoprotein. GGT, Gamma-glutamyl transferase. TNM, Tumor–Node–Metastasis

**Fig. 2:**
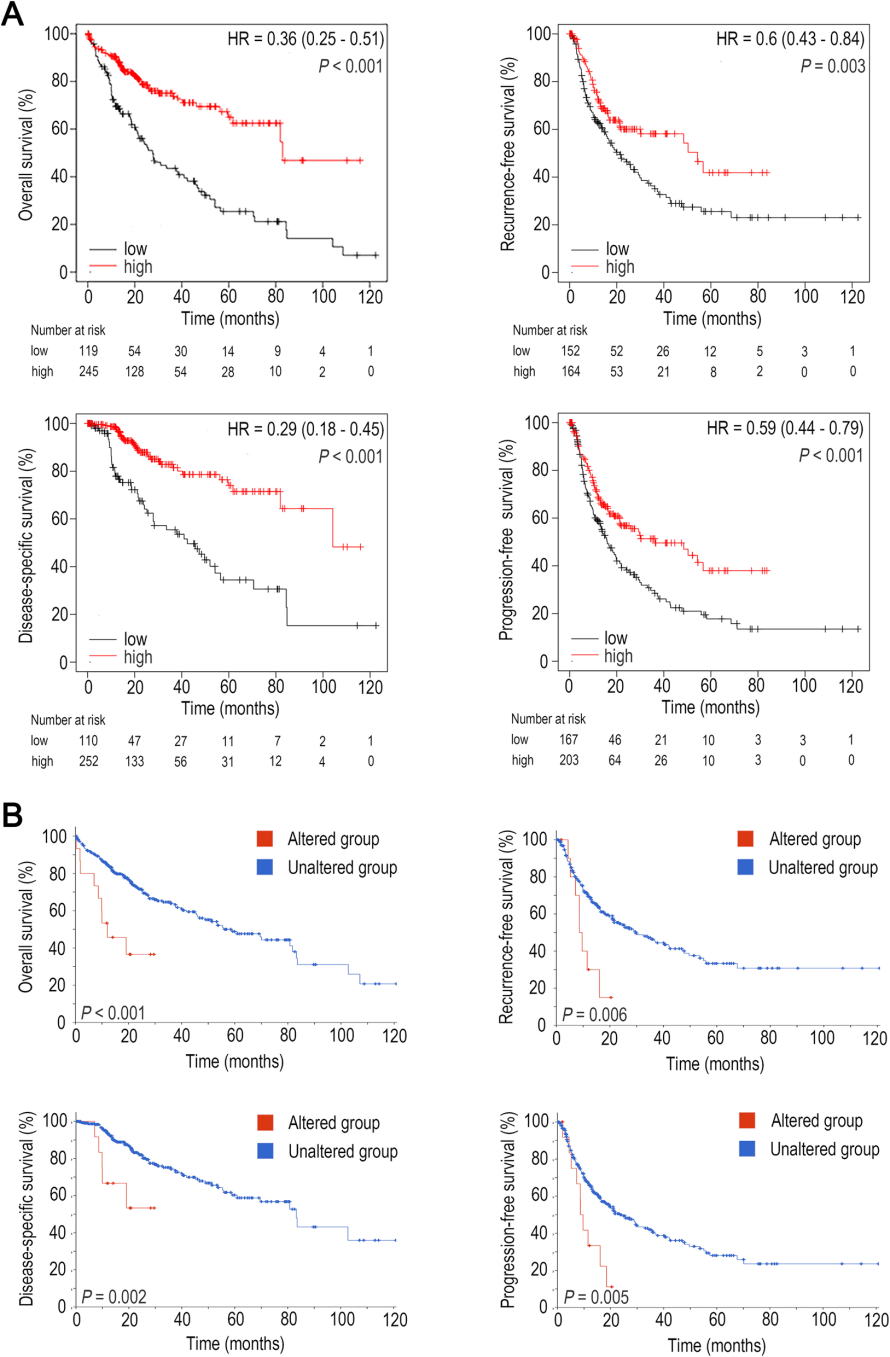
Prognostic value of DRD3 in HCC (Kaplan–Meier Plotter and cBioPortal). (**A**) Higher DRD3 mRNA expression was related to longer overall survival (OS), recurrence-free survival (RFS), disease-specific survival (DSS) and progression-free survival (PFS) times. (**B**) The DRD3 genetic alteration group had shorter OS, RFS, DSS and PFS times

Furthermore, online tools were used to confirm our results. Kaplan–Meier plotter analysis showed that higher expression of DRD3 mRNA was associated with better overall survival (OS, *P* < 0.001), recurrence-free survival (RFS, *P* = 0.003), disease-specific survival (DSS, *P* < 0.001), and progression-free survival (PFS, *P* < 0.001) (Fig. 2A). cBioPortal analysis revealed that the alteration group, which harbored genetic mutations in DRD3, exhibited poorer OS (*P* < 0.001), RFS (*P* = 0.006), DSS (*P* = 0.002) and PFS (*P* = 0.005) than the alteration-free group (Fig. 2B). Collectively, these results suggested that DRD3 can be an independent prognostic factor for HCC survival.

### DRD3 plays a vital role in HCC cell proliferation, migration and invasion in vitro

**Fig. 3:**
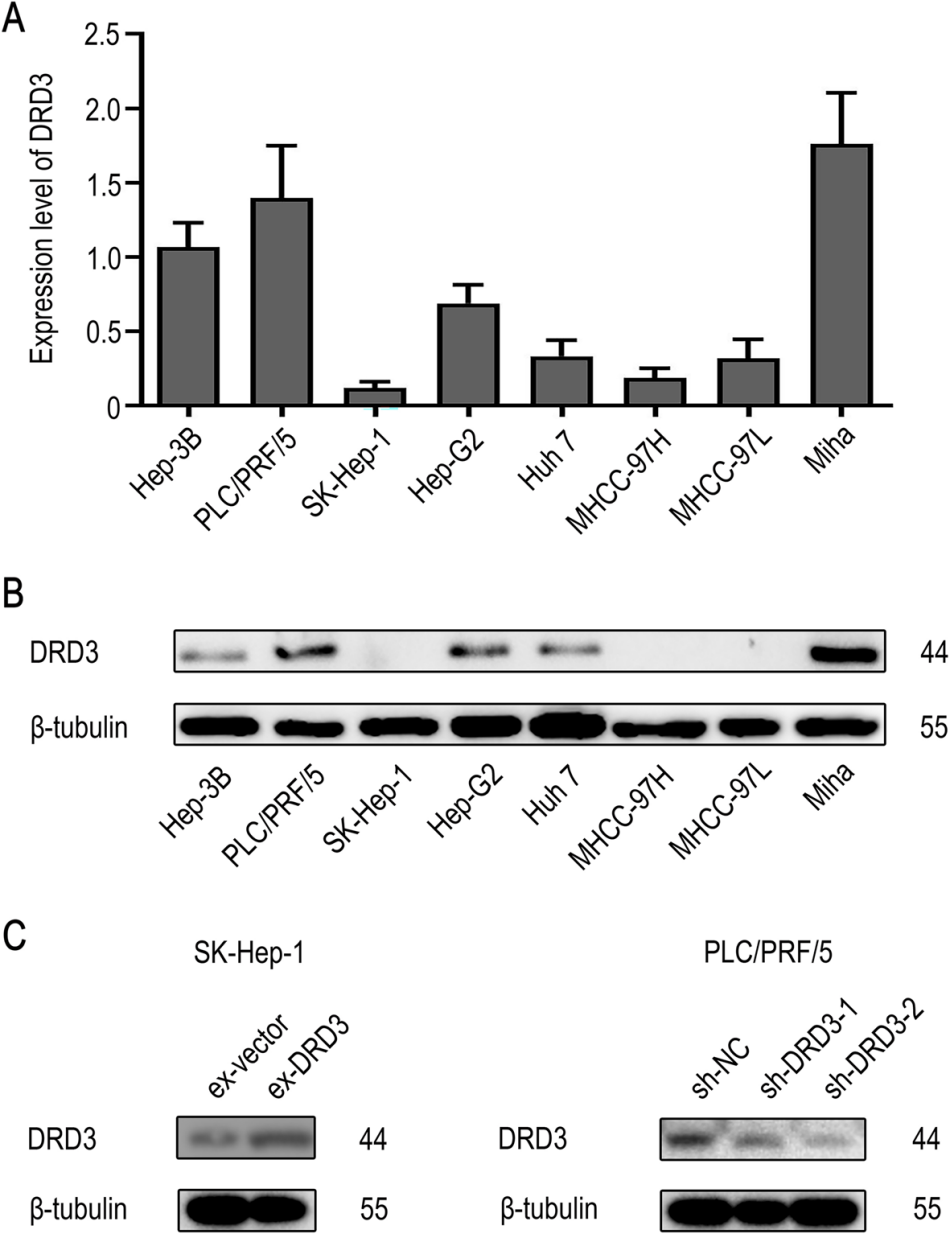
Expression of DRD3 in liver cell lines. (**A**, **B**) DRD3 mRNA and protein expression in HCC cell lines (MHCC-97H, MHCC-97L, Hep-3B, SK-Hep-1, PLC/PRF/5, Huh7 and Hep-G2) and a normal liver cell line (Miha). (**C**) DRD3 protein expression in the stably transfected cell lines. The blots showed in this figure were cropped from the original blots, the full-length blots are presented in Supplementary Fig. 2 and the cropped regions of the original blots were indicated by red boxes

DRD3 mRNA and protein expression was detected in HCC cell lines (Fig. 3A and 3B). Sh-DRD3-PLC/PRF/5 and ex-DRD3-SK-HEP-1 cell lines were established and verified (Fig. 3C) for subsequent experiments.

**Fig. 4:**
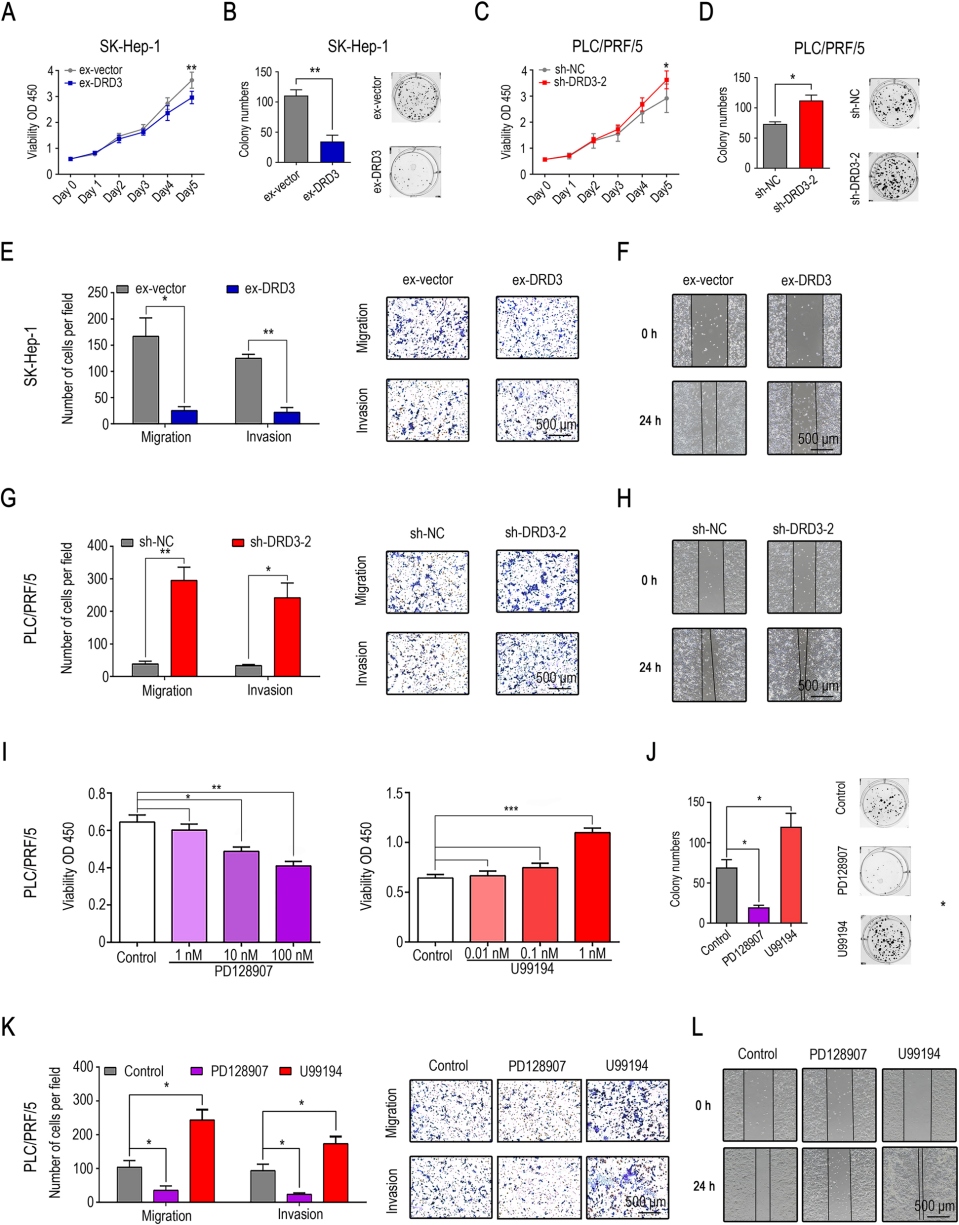
DRD3 plays a crucial role in HCC cell proliferation, migration and invasion in vitro. (**A**, **B**) CCK8 and colony formation assays demonstrated that upregulation of DRD3 expression significantly inhibited cell proliferation, and also suppressed the migration and invasion in HCC cells according to the Transwell assays and wound healing assays (**E**, **F**). (**C**, **D**, **G**, **H**) Downregulation of DRD3 expression promoted cell proliferation, migration and invasion. (**I**) PD128907 significantly suppressed cell proliferation at 10 nM and 100 nM. U99194 stimulated cell proliferation at 1 nM. (**J**) Colony number was decreased with PD128907 treating in HCC cells, and was increased with U99194 treating. (**K**, **L**) PD128907 (100 nM) inhibited migration and invasion in HCC cell lines, while U99194 (1 nM) promoted migration and invasion. * *P* < 0.05, ** *P* < 0.01, *** *P* < 0.001

The inhibitory effect of ex-DRD3-SK-HEP-1 on HCC cell proliferation was shown, while sh-DRD3-PLC/PRF/5 showed the opposite effect according to the CCK8 and colony formation assays (Fig. 4A, 4B, 4C and 4D). In the Transwell assays**,** higher numbers of migrated cells and invaded cells were observed in the sh-DRD3-PLC/PRF/5 cell groups, and ex-DRD3-SK-HEP-1 cells showed weaker migration and invasion behaviors (Fig. 4E and 4G), the wound healing assays had verified the same changes of migratory ability in stable transfected cell lines (Fig. 4F and 4H). Moreover, PD128907, a DRD3 agonist, suppressed proliferation, migration and invasion in HCC cell lines. In contrast, U99194, which is a DRD3 antagonist, enhanced proliferation, migration and invasion in the HCC cell lines (Fig. 4I, 4J, 4K and 4L). These results strongly suggested that DRD3 plays a vital role in HCC progression.

### DRD3 could suppress the cAMP/ERK/CREB pathway in HCC cell lines

**Fig. 5:**
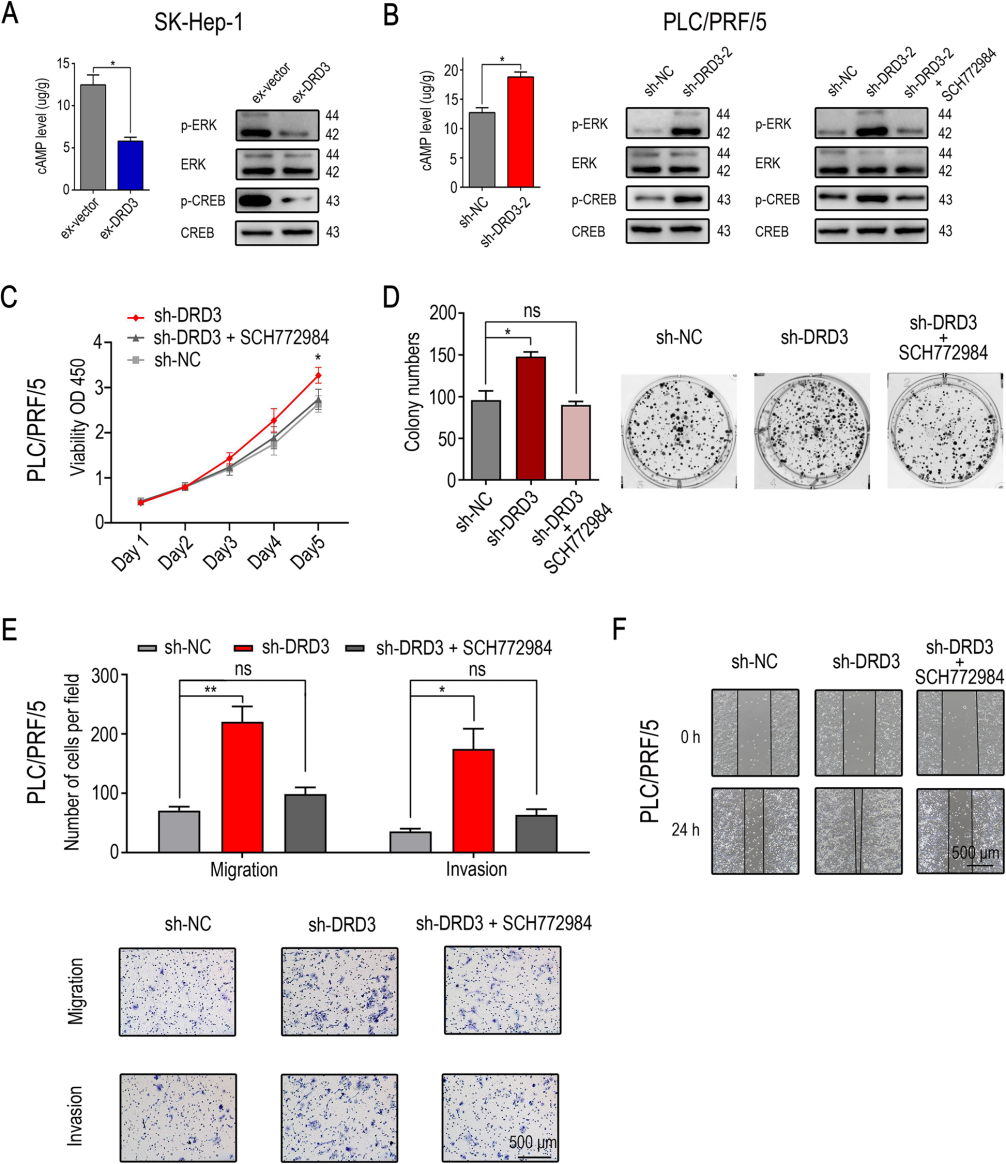
DRD3 may influence malignant biological behaviors of HCC cells via the cAMP/ERK/CREB pathway. (**A**, **B**) Overexpression of DRD3 decreased the levels of cAMP detected by ELISA, p-ERK and p-CREB detected by Western blot, while knock-down of DRD3 increased the levels of cAMP, p-ERK and p-CREB which could be reversed by treating with SCH772984 (1 µM). (**C**, **D**, **E**, **F**) SCH772984 (1 µM) abolished the effect of knock-down of DRD3 on the proliferation, migration and invasion of HCC cells. * *P* < 0.05, ** *P* < 0.01, *** *P* < 0.001. The blots showed in this figure were cropped from the original blots, the full-length blots are presented in Supplementary Fig. 3 and the cropped regions of the original blots were indicated by red boxes

To determine the mechanism underlying the effect of DRD3 on proliferation, migration and invasion in HCC cell lines, more experiments were performed. From the KEGG database, we found that DRD3 can suppress the cAMP/ERK/CREB pathway in the nervous system (Supplementary Fig. 1A). Western blot analysis and ELISA revealed that knock-down of DRD3 increased the levels of cAMP detected, p-ERK and p-CREB detected; on the other hand, overexpression of DRD3 decreased the levels of cAMP, p-ERK and p-CREB (Fig. 5A and 5B). Thus, we hypothesized that DRD3 might influence malignant biological behaviors of HCC cells via the cAMP/ERK/CREB pathway. SCH772984, an ERK antagonist, was used to verify our hypothesis. SCH772984 abolished the effect of knock-down of DRD3 on malignant biological behaviors including the proliferation, migration and invasion of HCC cells (Fig. 5C, 5D, 5E and 5F), and the activation of the cAMP/ERK/CREB pathway changed correspondingly (Fig. 5B).

### Knock-down of DRD3 could promote tumor growth in vivo

**Fig. 6:**
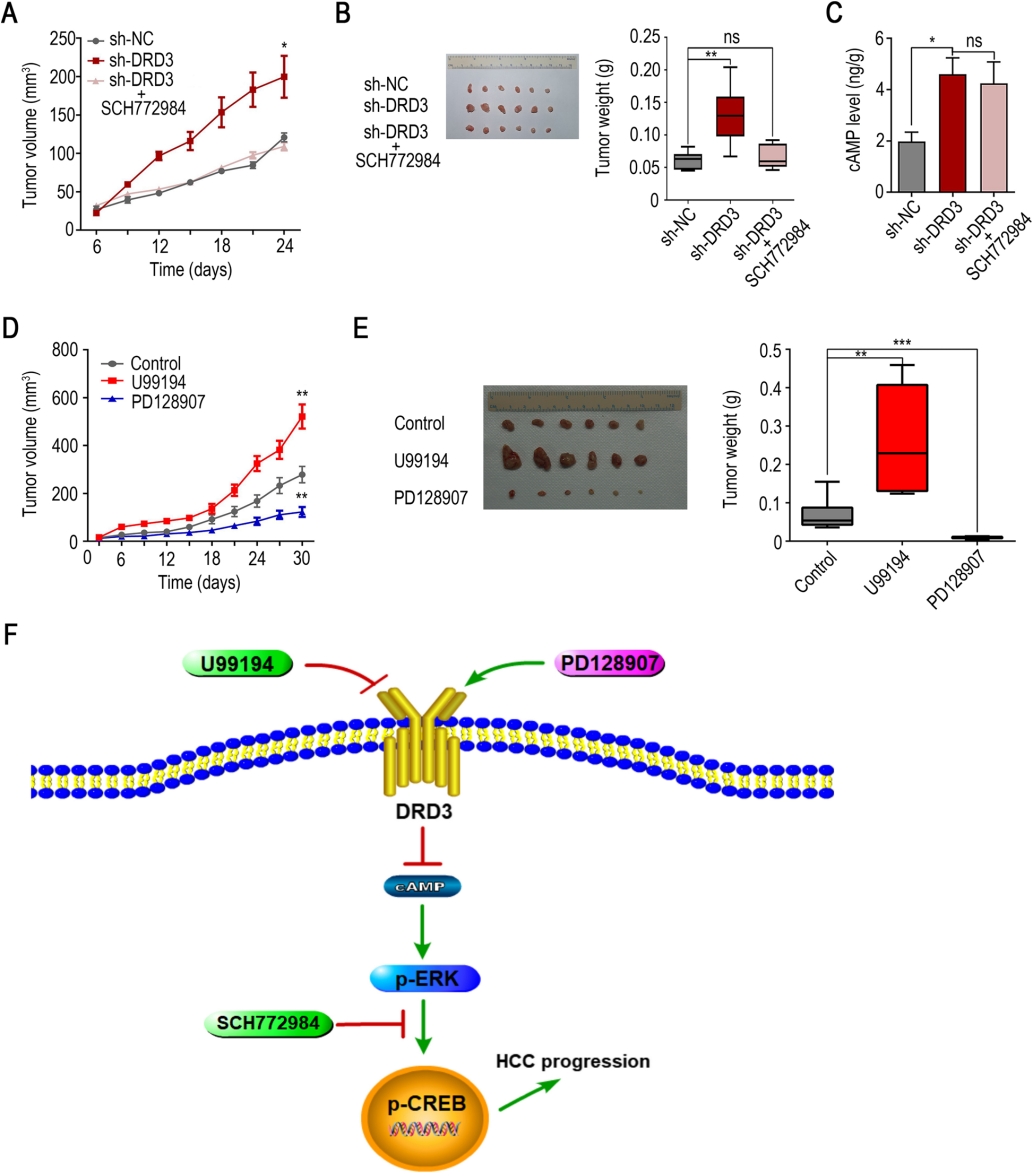
The Effect of DRD3 on tumor growth in vivo. (**A**, **B**) Knock-down of DRD3 could promote tumor growth in vivo, and SCH772984 could also abolished this effect in vivo. (**C**) The cAMP level of three groups of tumors. (**D,** **E**) PD128907 suppressed tumor growth in vivo, and U99194 promoted tumor growth in vivo. (F) Model illustrating possible mechanisms by which the loss of DRD3 contributes to HCC progression. * *P* < 0.05, ** *P* < 0.01, *** *P* < 0.001

Mouse xenograft models were established to verify the role of DRD3 in HCC. Based on the outcome, knock-down of DRD3 could promote tumor growth in vivo, and SCH772984 could also abolished this effect in vivo (Fig. 6A and 6B). The loss of DRD3 could increase the level of cAMP in tumors, and SCH772984 could not change this phenomenon (Fig. 6C). Furthermore, both the volume and weight of tumors were lower after treatment with PD128907 than in the control group. After treatment with U99194, tumor growth was enhanced (Fig. 6D and 6E). These results prompted us to conclude that DRD3 agonist treatment can suppress tumor growth in vivo, which meant that DRD3 might be a potential therapeutic target for HCC.

## Discussion

As reported, dopamine receptors are associated with proliferation and metastasis in a wide variety of tumors, such as lung cancer, breast cancer and gastric carcinoma. However, there are little research related to the effect of DRD3 in HCC cancer progression, while numerous studies about the carcinogenesis of DRD1 and DRD2 have been published [31–33], and tumor-related research on DRD3 is rare. To our knowledge, no research on DRD3 in HCC has been reported. This study first attempted to observe the relationship between DRD3 expression and the prognosis of patients with HCC; furthermore, the effects of DRD3 on HCC cell proliferation and metastasis were tested, and we tried to explore the possible mechanism.

DRD3 had been reported that its expression was lower in cholangiocarcinoma cell lines SG231 and CCLP-1 [16], which was consisted with our finding. But we had found that DRD3 expression was lower in HCC tissues (Fig. 1A), which is in contradiction to Akbari’s findings in breast cancer [15]. However, the samples of Akbari’s research were blood samples in breast patients, not the breast cancer tissues. Furthermore, the same characteristic didn’t always play the same role in different cancer type. For example, prolactin [34] could prevent hepatocellular carcinoma, but in breast cancer, prolactin could promote cancer progression [35]. DRD3 might also play different role in different cancer. And the result of DRD3 expression in GSE14520 (Fig. 1B) had verified our find too.

The results of immunohistochemical staining and Kaplan–Meier analysis showed that HCC patients with high DRD3 protein expression had significantly better RFS and OS compared with the patients with lower DRD3 protein expression (*P* < 0.05), and the multivariate Cox regression analysis results suggested that DRD3 is an independent factor related to the RFS and OS of the HCC patients (*P* < 0.05). Based on the results from the online tools, we found that the mRNA expression of DRD3 was associated with RFS, OS, PFS and DSS in HCC patients, while DRD3 gene mutation was also related to the survival of patients (*P* < 0.05). Collectively, these results indicated that DRD3 has definite clinical value in predicting the prognosis of HCC patients. The real-time qPCR results showed that DRD3 mRNA expression in HCC tissues was lower than that in adjacent nontumor tissues and that DRD3 mRNA expression was higher in the noncancerous cell line Miha than in HCC cell lines, suggesting that DRD3 may be a potential antitumor therapeutic target in HCC. The results of cell proliferation, migration, and invasion experiments both in vivo and in vitro supported our hypothesis.

By querying the KEGG pathway database, we found that DRD3 in the nervous system can inhibit adenylate cyclase, which can cause a reduction in cAMP. cAMP, a second messenger in signal transmission, can regulate MAPK signaling pathways, ultimately affecting various behavioral characteristics of cells [36]. ERK is an important member of the MAPK family, and the MAPK signaling pathway mediates a variety of biological characteristics of cells, including tumor cell proliferation, apoptosis, migration, invasion and vascular survival behavior [37, 38]. CREB is an important transcription factor that may play an extensive role in the multifaceted pathophysiology of cancer. Activation of CREB promotes cell proliferation and enhances cell migration and invasion [39]. Modification of DRD3 function could modulate the malignant biological behaviors of HCC cells by targeting the cAMP/ERK/CREB pathway (Fig. 6F). It had been reported that DRD3 was involved in the process of autophagy in HeLa cell line [40], DRD3 may also induce autophagy in HCC cells. However, the pathogenesis of cancer is complex, and further studies are needed to clarify the whole mechanism.

In summary, high expression of DRD3 in HCC tissue is correlated with a better prognosis for patients, and DRD3 may become a molecular marker to independently predict the survival of patients. Agonists or antagonists of DRD3 respectively suppressed or enhanced the proliferation, migration and invasion of HCC cells by regulating the cAMP/ERK/CREB signaling pathway, and the corresponding results were obtained in animal experiments, indicating that DRD3 may be a molecular therapeutic target in hepatocellular carcinoma. The results of this study may provide new treatment approaches for HCC patients.

## Supplementary Information

**Additional file 1.**

**Additional file 2.**

**Additional file 3.** 

**Additional file 4.** 

## Data Availability

The raw data of the experiment had been uploaded onto the Research Data Deposit (RDD) (https://www.researchdata.org.cn/default.aspx) with an RDD number of RDDB2022527925.

## References

[1] Yang JD, Hainaut P, Gores GJ, Amadou A, Plymoth A, Roberts LR (2019). A global view of hepatocellular carcinoma: trends, risk, prevention and management. Nat Rev Gastroenterol Hepatol.

[2] Collaborators GBDCoD (2018). Global, regional, and national age-sex-specific mortality for 282 causes of death in 195 countries and territories, 1980–2017: a systematic analysis for the Global Burden of Disease Study 2017. Lancet.

[3] Leung CY, Huang HL, Rahman MM, Nomura S, Krull Abe S, Saito E, Shibuya K (2020). Cancer incidence attributable to tuberculosis in 2015: global, regional, and national estimates. BMC Cancer.

[4] Fitzmaurice C, Abate D, Abbasi N, Abbastabar H, Abd-Allah F, Abdel-Rahman O, Abdoli A, Abdollahpour I, Global Burden of Disease Cancer C (2019). Global, Regional, and National Cancer Incidence, Mortality, Years of Life Lost, Years Lived With Disability, and Disability-Adjusted Life-Years for 29 Cancer Groups, 1990 to 2017: A Systematic Analysis for the Global Burden of Disease Study. JAMA Oncol.

[5] Molla MD, Dessie G, Akalu Y, Ayelign B (2020). Hepatocellular Expression of SIRT1 and Its Effect on Hepatocellular Carcinoma Progression: A Future Therapeutic Perspective. Int J Hepatol.

[6] Baxter MA, Glen H, Evans TR (2018). Lenvatinib and its use in the treatment of unresectable hepatocellular carcinoma. Future Oncol.

[7] Dong Y, Liu TH, Yau T, Hsu C. Novel systemic therapy for hepatocellular carcinoma. Hepatol Int. 2020;14(5):638-651. 10.1007/s12072-020-10073-7 32661949

[8] Gingrich JA, Caron MG (1993). Recent advances in the molecular biology of dopamine receptors. Annu Rev Neurosci.

[9] Wang X, Wang ZB, Luo C, Mao XY, Li X, Yin JY, Zhang W, Zhou HH, Liu ZQ (2019). The Prospective Value of Dopamine Receptors on Bio-Behavior of Tumor. J Cancer.

[10] Yan Y, Pan J, Chen Y, Xing W, Li Q, Wang D, Zhou X, Xie J, Miao C, Yuan Y (2020). Increased dopamine and its receptor dopamine receptor D1 promote tumor growth in human hepatocellular carcinoma. Cancer Commun.

[11] Borcherding DC, Tong W, Hugo ER, Barnard DF, Fox S, LaSance K, Shaughnessy E, Ben-Jonathan N (2016). Expression and therapeutic targeting of dopamine receptor-1 (D1R) in breast cancer. Oncogene.

[12] Sachlos E, Risueno RM, Laronde S, Shapovalova Z, Lee JH, Russell J, Malig M, McNicol JD, Fiebig-Comyn A, Graham M (2012). Identification of drugs including a dopamine receptor antagonist that selectively target cancer stem cells. Cell.

[13] Yin T, He S, Shen G, Ye T, Guo F, Wang Y (2015). Dopamine receptor antagonist thioridazine inhibits tumor growth in a murine breast cancer model. Mol Med Rep.

[14] Jandaghi P, Najafabadi HS, Bauer AS, Papadakis AI, Fassan M, Hall A, Monast A, von Knebel DM, Neoptolemos JP, Costello E (2016). Expression of DRD2 Is Increased in Human Pancreatic Ductal Adenocarcinoma and Inhibitors Slow Tumor Growth in Mice. Gastroenterology.

[15] Akbari ME, Kashani FL, Ahangari G, Pornour M, Hejazi H, Nooshinfar E, Kabiri M, Hosseini L (2016). The effects of spiritual intervention and changes in dopamine receptor gene expression in breast cancer patients. Breast Cancer.

[16] Coufal M, Invernizzi P, Gaudio E, Bernuzzi F, Frampton GA, Onori P, Franchitto A, Carpino G, Ramirez JC, Alvaro D (2010). Increased local dopamine secretion has growth-promoting effects in cholangiocarcinoma. Int J Cancer.

[17] Zhang QB, Zhang BH, Zhang KZ, Meng XT, Jia QA, Zhang QB, Bu Y, Zhu XD, Ma DN, Ye BG (2016). Moderate swimming suppressed the growth and metastasis of the transplanted liver cancer in mice model: with reference to nervous system. Oncogene.

[18] Dumas J, Gargano MA, Dancik GM (2016). shinyGEO: a web-based application for analyzing gene expression omnibus datasets. Bioinformatics.

[19] Roessler S, Jia HL, Budhu A, Forgues M, Ye QH, Lee JS, Thorgeirsson SS, Sun Z, Tang ZY, Qin LX (2010). A unique metastasis gene signature enables prediction of tumor relapse in early-stage hepatocellular carcinoma patients. Cancer Res.

[20] Menyhart O, Nagy A, Gyorffy B (2018). Determining consistent prognostic biomarkers of overall survival and vascular invasion in hepatocellular carcinoma. R Soc Open Sci.

[21] Gao J, Aksoy BA, Dogrusoz U, Dresdner G, Gross B, Sumer SO, Sun Y, Jacobsen A, Sinha R (2013). Larsson E *et al*: Integrative analysis of complex cancer genomics and clinical profiles using the cBioPortal. Sci Signal..

[22] Chen DT, Pan JH, Chen YH, Xing W, Yan Y, Yuan YF, Zeng WA (2019). The mu-opioid receptor is a molecular marker for poor prognosis in hepatocellular carcinoma and represents a potential therapeutic target. Br J Anaesth.

[23] Kanehisa M, Furumichi M, Sato Y, Ishiguro-Watanabe M, Tanabe M (2021). KEGG: integrating viruses and cellular organisms. Nucleic Acids Res.

[24] Wu F, Zhang FY, Tan GQ, Chen WJ, Huang B, Yan L, Zhang HL, Chen S, Jiao Y, Wang BL (2021). Down-regulation of EGFL8 regulates migration, invasion and apoptosis of hepatocellular carcinoma through activating Notch signaling pathway. BMC Cancer.

[25] Yang YY, Yu HH, Jiao XL, Li LY, Du YH, Li J, Lv QW, Zhang HN, Zhang J, Hu CW (2021). Angiopoietin-like proteins 8 knockout reduces intermittent hypoxia-induced vascular remodeling in a murine model of obstructive sleep apnea. Biochem Pharmacol.

[26] Shrestha R, Bridle KR, Crawford DHG, Jayachandran A (2021). Immune checkpoint molecules are regulated by transforming growth factor (TGF)-beta1-induced epithelial-to-mesenchymal transition in hepatocellular carcinoma. Int J Med Sci.

[27] Hong J, Hu K, Yuan Y, Sang Y, Bu Q, Chen G, Yang L, Li B, Huang P, Chen D (2012). CHK1 targets spleen tyrosine kinase (L) for proteolysis in hepatocellular carcinoma. J Clin Invest.

[28] Kilkenny C, Browne WJ, Cuthill IC, Emerson M, Altman DG (2012). Improving bioscience research reporting: the ARRIVE guidelines for reporting animal research. Osteoarthritis Cartilage.

[29] Xing W, Chen DT, Pan JH, Chen YH, Yan Y, Li Q, Xue RF, Yuan YF, Zeng WA (2017). Lidocaine Induces Apoptosis and Suppresses Tumor Growth in Human Hepatocellular Carcinoma Cells In Vitro and in a Xenograft Model In Vivo. Anesthesiology.

[30] Chen D, Chen Y, Yan Y, Pan J, Xing W, Li Q, Zeng W (2017). Down-regulation of the tumour suppressor kappa-opioid receptor predicts poor prognosis in hepatocellular carcinoma patients. BMC Cancer.

[31] Kline CLB, Ralff MD, Lulla AR, Wagner JM, Abbosh PH, Dicker DT, Allen JE, El-Deiry WS (2018). Role of Dopamine Receptors in the Anticancer Activity of ONC201. Neoplasia.

[32] Peverelli E, Giardino E, Treppiedi D, Meregalli M, Belicchi M, Vaira V, Corbetta S, Verdelli C, Verrua E, Serban AL (2017). Dopamine receptor type 2 (DRD2) and somatostatin receptor type 2 (SSTR2) agonists are effective in inhibiting proliferation of progenitor/stem-like cells isolated from nonfunctioning pituitary tumors. Int J Cancer.

[33] Cherubini E, Di Napoli A, Noto A, Osman GA, Esposito MC, Mariotta S, Sellitri R, Ruco L, Cardillo G, Ciliberto G (2016). Genetic and Functional Analysis of Polymorphisms in the Human Dopamine Receptor and Transporter Genes in Small Cell Lung Cancer. J Cell Physiol.

[34] Hartwell HJ, Petrosky KY, Fox JG, Horseman ND, Rogers AB (2014). Prolactin prevents hepatocellular carcinoma by restricting innate immune activation of c-Myc in mice. Proc Natl Acad Sci U S A.

[35] Kavarthapu R, Anbazhagan R, Dufau ML. Crosstalk between PRLR and EGFR/HER2 Signaling Pathways in Breast Cancer. Cancers (Basel). 2021;13(18):4685. 10.3390/cancers13184685 PMC8467304 34572912

[36] Maity S, Chandanathil M, Millis RM, Connor SA. Norepinephrine stabilizes translation-dependent, homosynaptic long-term potentiation through mechanisms requiring the cAMP sensor Epac, mTOR and MAPK. Eur J Neurosci. 2020;52(7):3679-3688. 10.1111/ejn.14735 32275785

[37] Eblen ST (2018). Extracellular-Regulated Kinases: Signaling From Ras to ERK Substrates to Control Biological Outcomes. Adv Cancer Res.

[38] McCubrey JA, Steelman LS, Chappell WH, Abrams SL, Wong EW, Chang F, Lehmann B, Terrian DM, Milella M, Tafuri A (2007). Roles of the Raf/MEK/ERK pathway in cell growth, malignant transformation and drug resistance. Biochim Biophys Acta.

[39] Steven A, Seliger B (2016). Control of CREB expression in tumors: from molecular mechanisms and signal transduction pathways to therapeutic target. Oncotarget.

[40] Li Z, Ji X, Wang W, Liu J, Liang X, Wu H, Liu J, Eggert US, Liu Q, Zhang X (2016). Ammonia Induces Autophagy through Dopamine Receptor D3 and MTOR. PLoS ONE.

