# Supplementary material for: Dopamine receptor D3 is related to prognosis in human hepatocellular carcinoma and inhibits tumor growth
Source: BMC Cancer. 2022 Dec 2;22:1248. doi: 10.1186/s12885-022-10368-y (PMC9717446; doi:10.1186/s12885-022-10368-y)
Supplement: Supplementary file 1 — Additional file 1. [file 12885_2022_10368_MOESM1_ESM.docx]

A


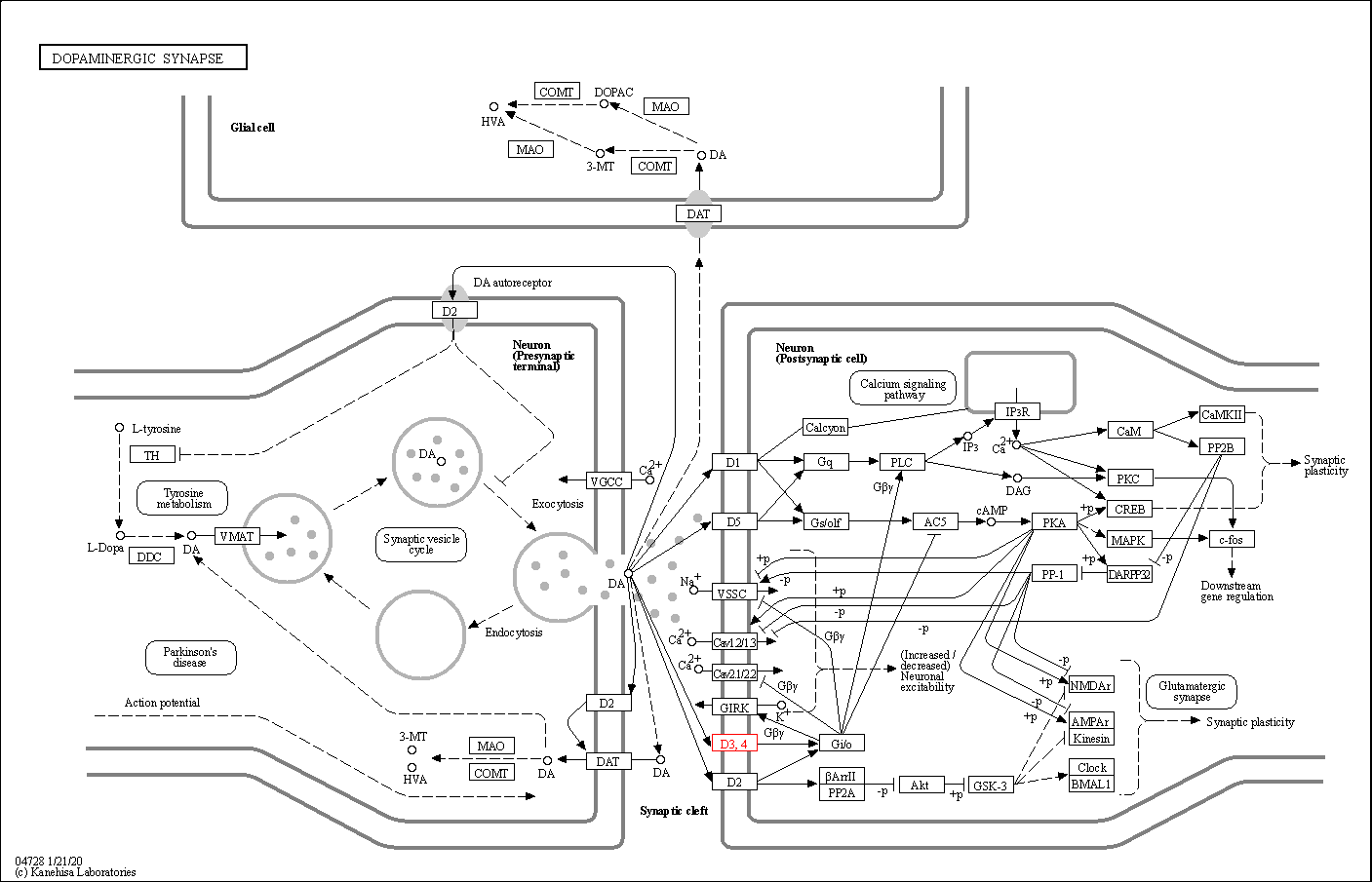


**Supplementary figure 1. The pathway map04728 in KEGG database.** (A) The pathways in dopaminergic synapse.
