# Supplementary material for: Dopamine receptor D3 is related to prognosis in human hepatocellular carcinoma and inhibits tumor growth
Source: BMC Cancer. 2022 Dec 2;22:1248. doi: 10.1186/s12885-022-10368-y (PMC9717446; doi:10.1186/s12885-022-10368-y)
Supplement: Supplementary file 2 — Additional file 2. [file 12885_2022_10368_MOESM2_ESM.doc]

Supplementary figure 2. Full blots related to Fig.3B and 3C in manuscript.


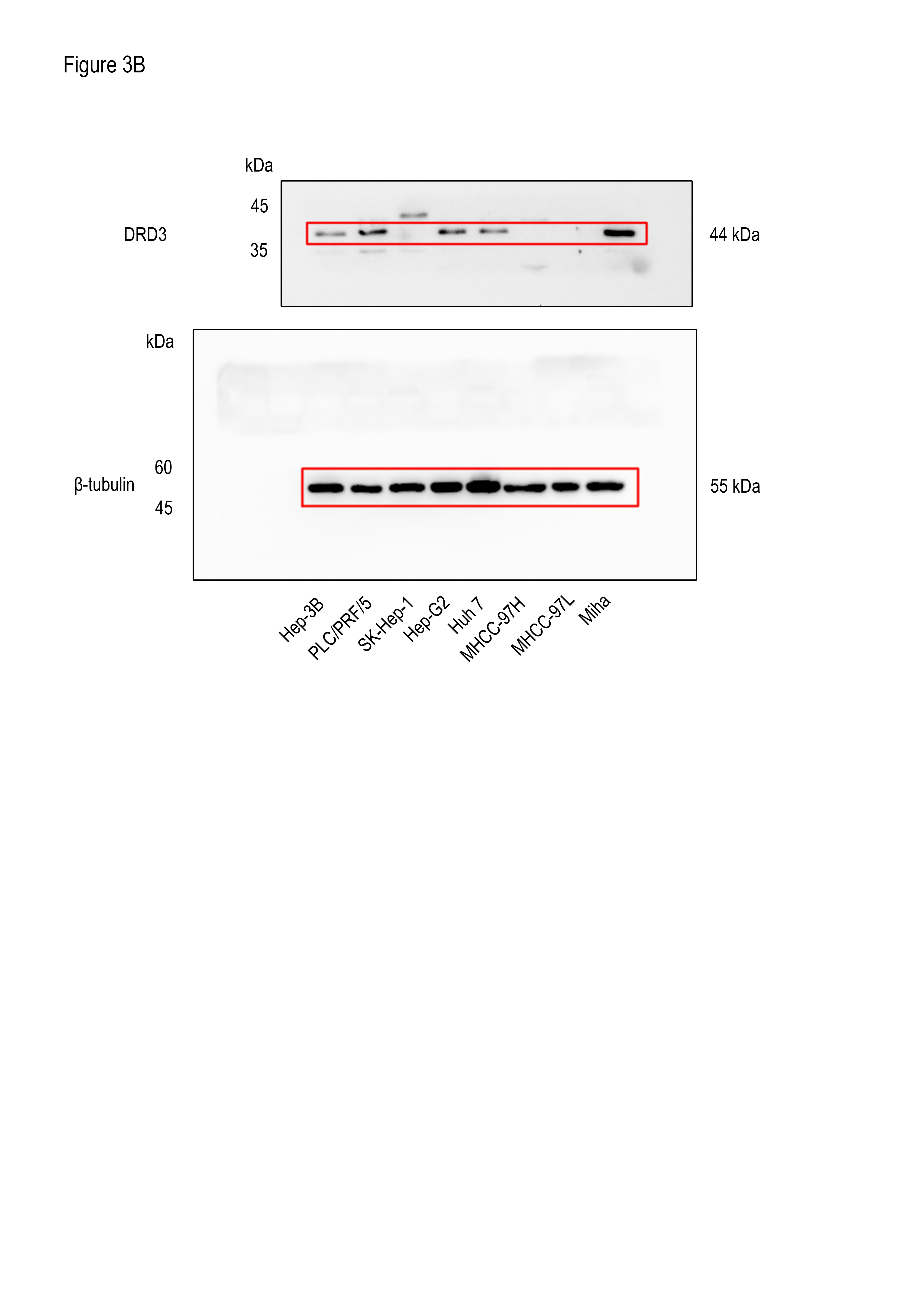

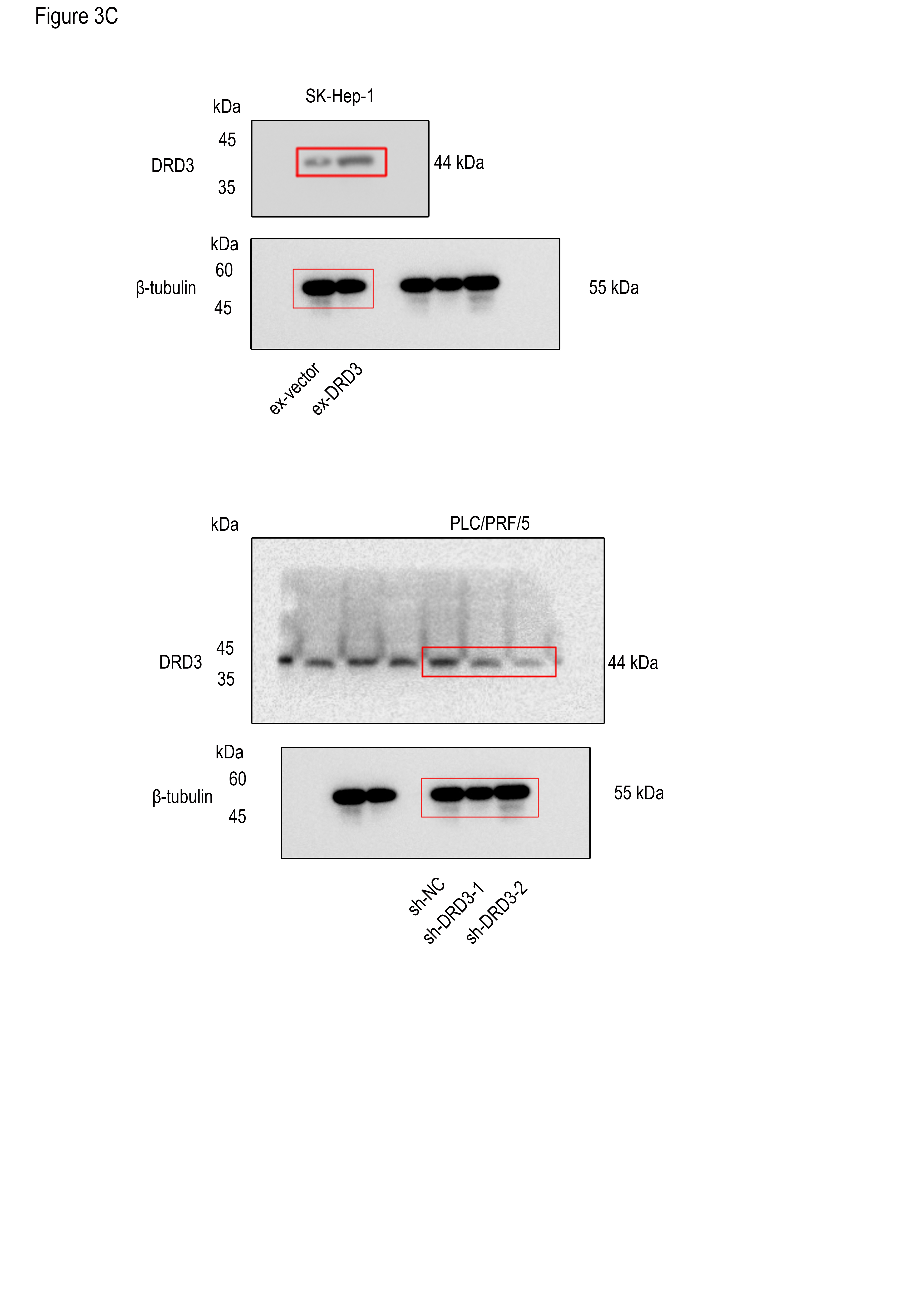


The blots in the red boxes were croped and presented in manuscript figure 3.

A explaination was mentioned in the figure legend of figure 3.

Supplementary figure 3. Full blots related to Fig.5A and 5B in manuscript.


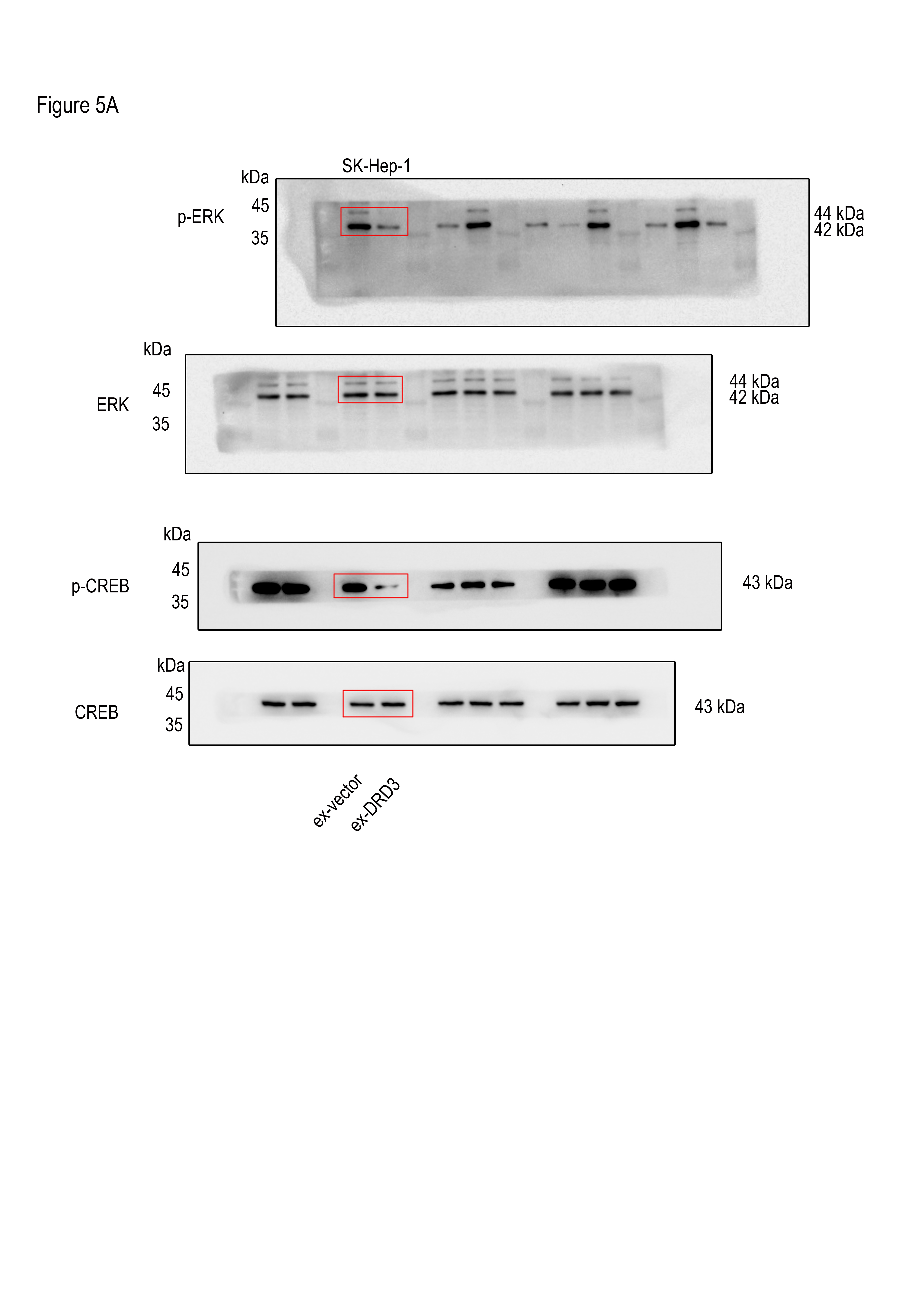


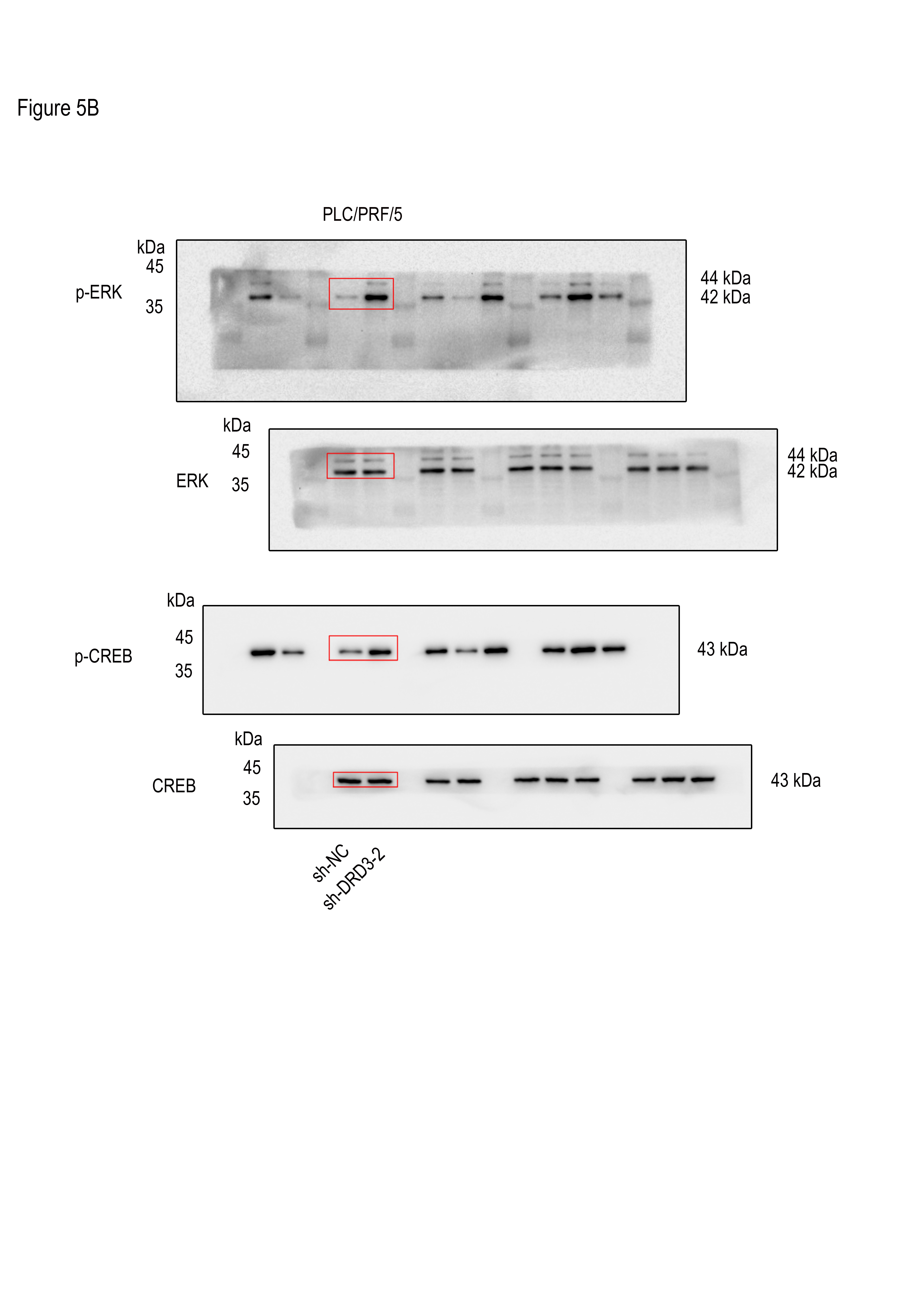


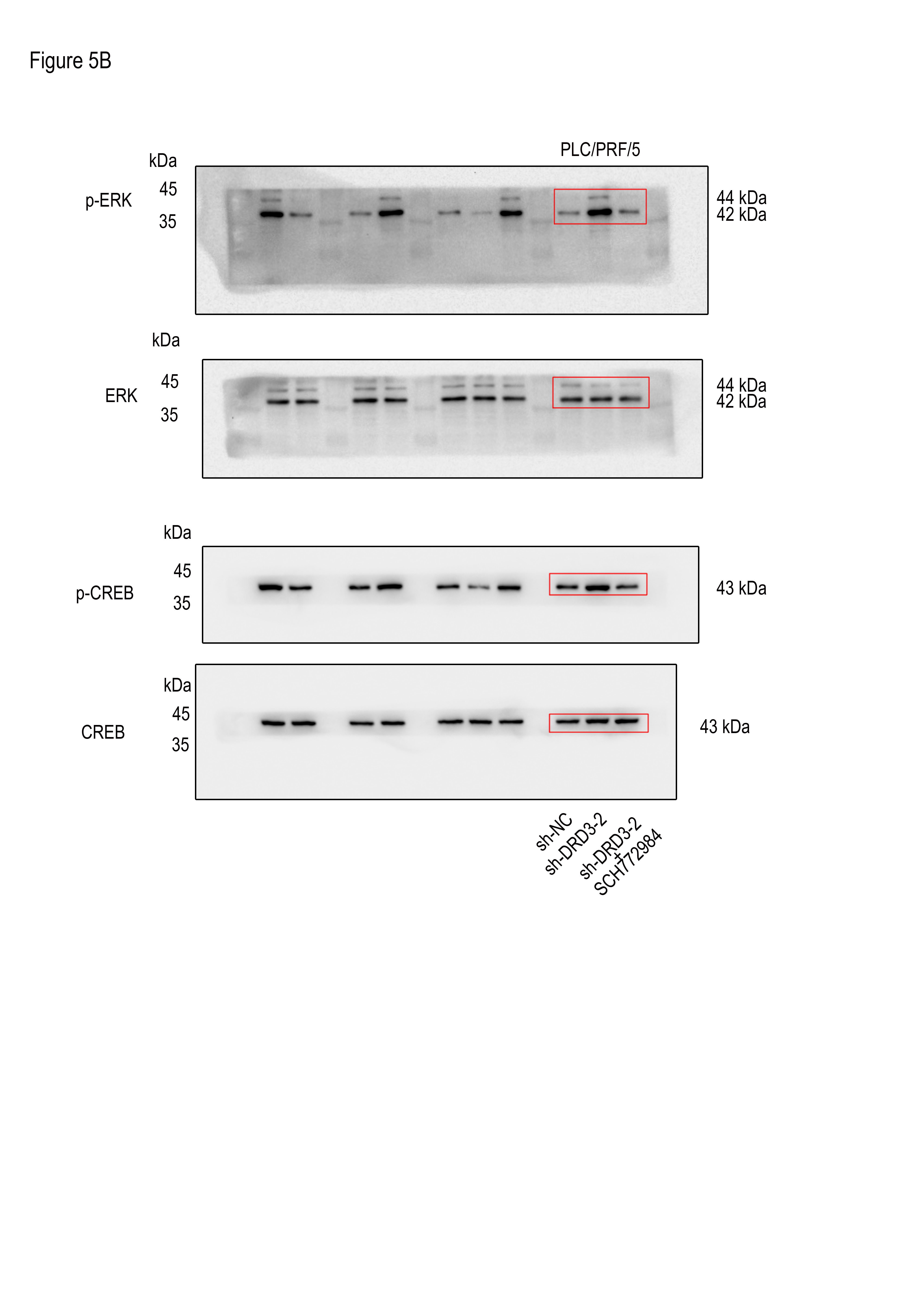


The blots in the red boxes were croped and presented in manuscript figure 5.

A explaination was mentioned in the figure legend of figure 5.
