# Supplementary material for: Dopamine receptor D3 is related to prognosis in human hepatocellular carcinoma and inhibits tumor growth
Source: BMC Cancer. 2022 Dec 2;22:1248. doi: 10.1186/s12885-022-10368-y (PMC9717446; doi:10.1186/s12885-022-10368-y)
Supplement: Supplementary file 3 — Additional file 3. [file 12885_2022_10368_MOESM3_ESM.doc]

**Supplementary files online**

**1. Supplementary Table 1.** Primer sequences used in the Real time qPCR.

**2. Supplementary Table 2.** Correlation of DRD3 expression with clinicopathologic characteristics in 218 patients with HCC.

**1. Supplementary Table 1. Primer sequences used in the Real time qPCR**

| Locus | Primers |
| --- | --- |
| GADPH- F | 5’-AGAAGGCTGGGGCTCATTTG-3’ |
| GADPH -R | 5’-GAGGGGCCATCCACAGTCTTC-3’ |
| DRD3-F | 5’- GTCCTTGTCTATGCCAGAATCTATG -3’ |
| DRD3-R | 5’- TCTTGGAAGCCTGGTCCAC -3’ |

**2. Supplementary Table 2. Correlation of** **DRD3 expression with clinicopathologic characteristics in 218 patients with HCC^1^**

| **Characteristics** | **Cases** | **DRD3 protein** | | |
| --- | --- | --- | --- | --- |
|  |  | **Low expression** | **High expression** | ***P* Value** |
| Gender |  |  |  |  |
| Female | 31 | 18 (58.1%) | 13 (41.9%) |  |
| Male | 187 | 87 (46.5%) | 100 (53.5%) | 0.234 |
| Age (y) |  |  |  |  |
| ≤ 50 | 117 | 52 (44.4%) | 65 (55.6%) |  |
| > 50 | 101 | 53 (52.5%) | 48 (47.5%) | 0.237 |
| HbsAg |  |  |  |  |
| Negative | 30 | 19 (42.3%) | 11 (57.7%) |  |
| Positive | 188 | 86 (49.0%) | 102 (51.0%) | 0.073 |
| Child-Pugh classification**^2^** | |  |  |  |
| A | 215 | 103 (47.9%) | 112 (52.1%) |  |
| B | 3 | 2(66.7%) | 1 (33.3%) | 0.518 |
| AFP (ng/ml) |  |  |  |  |
| ≤ 20 | 77 | 35 (45.5%) | 42 (54.5%) |  |
| 20-400 | 52 | 23 (43.2%) | 29 (55.8%) |  |
| >400 | 89 | 47 (52.8%) | 42 (47.2%) | 0.517 |
| GGT (units/L) |  |  |  |  |
| ≤ 50 | 104 | 50 (48.1%) | 54 (51.9%) |  |
| > 50 | 114 | 55 (48.2%) | 59 (51.8%) | 0.980 |
| Tumor size (cm) |  |  |  |  |
| ≤ 5 | 106 | 45 (42.5%) | 61 (57.5%) |  |
| > 5 | 112 | 60 (53.6%) | 52 (46.4%) | 0.101 |
| Satellite nodule | |  |  |  |
| No/incomplete | 184 | 88 (47.8%) | 96 (52.2%) |  |
| Yes | 34 | 17 (50.0%) | 17 (50.0%) | 0.816 |
|  | | | | |
| (Continued) |  |  | |  |
| **Characteristics** | **Cases** | **DRD3 protein** | | ***P* Value** |
|  |  | **Low expression** | **High expression** |  |
| Tumor capsule |  |  |  |  |
| No | 67 | 34 (50.7%) | 33 (49.3%) |  |
| Yes | 151 | 71(47.0%) | 80 (53.0%) | 0.611 |
| Vascular invasion |  |  |  |  |
| No | 191 | 87 (45.5%) | 104 (54.5%) |  |
| Yes | 27 | 18 (66.7%) | 9 (33.3%) | **0.040** |
| Cirrhosis |  |  |  |  |
| No | 39 | 20 (51.3%) | 19 (48.7%) |  |
| Yes | 179 | 85 (47.5%) | 94 (52.5%) | 0.667 |
| TNM classification  46 | |  |  |  |
| I/ II | 180 | 81 (45.0%) | 99 (55.0%) |  |
| III /IV | 38 | 24 (63.2%) | 14 (36.8%) | **0.042** |

**^1^**Values of statistical significance are in bold.

**^2^**There is no patient with Child-Pugh Class C.
