# Supplementary material for: Dopamine receptor D3 is related to prognosis in human hepatocellular carcinoma and inhibits tumor growth
Source: BMC Cancer. 2022 Dec 2;22:1248. doi: 10.1186/s12885-022-10368-y (PMC9717446; doi:10.1186/s12885-022-10368-y)

The specific detection of antibodies (The samples were the protein of HCC cell line including PLC/PRF/5, Huh7 and Hep-G2)：

DRD3:


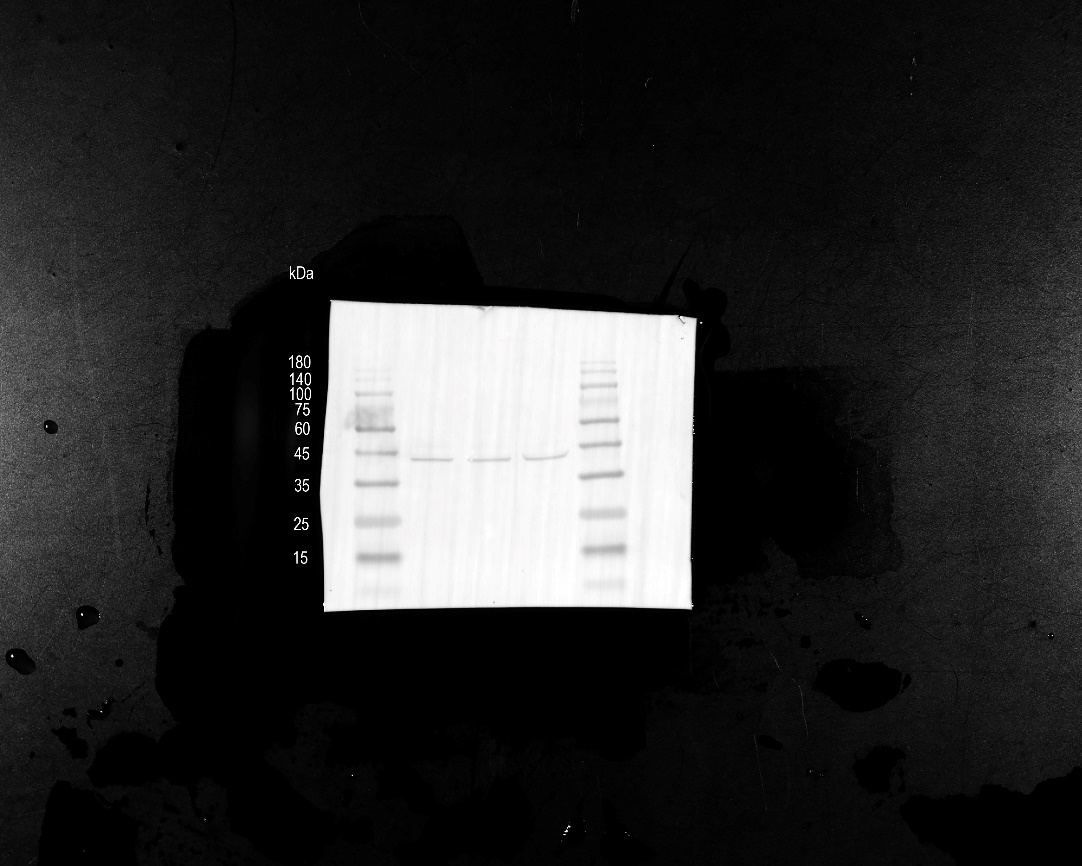


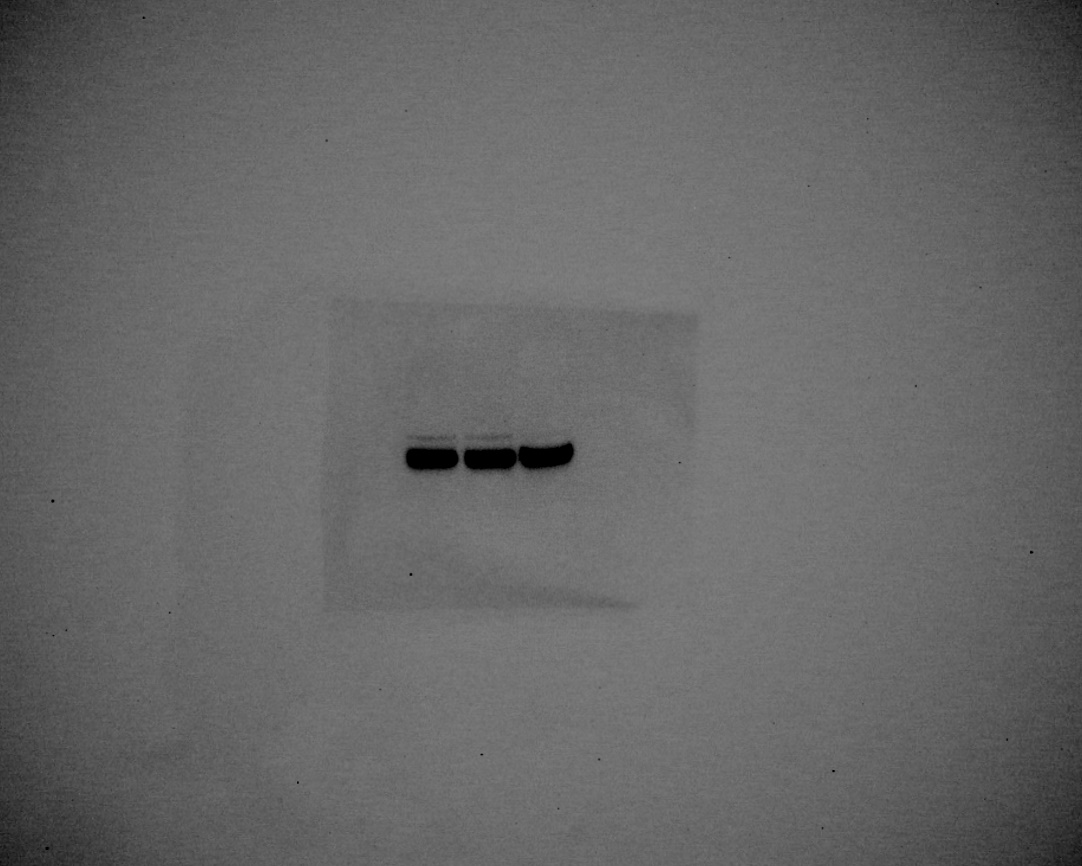

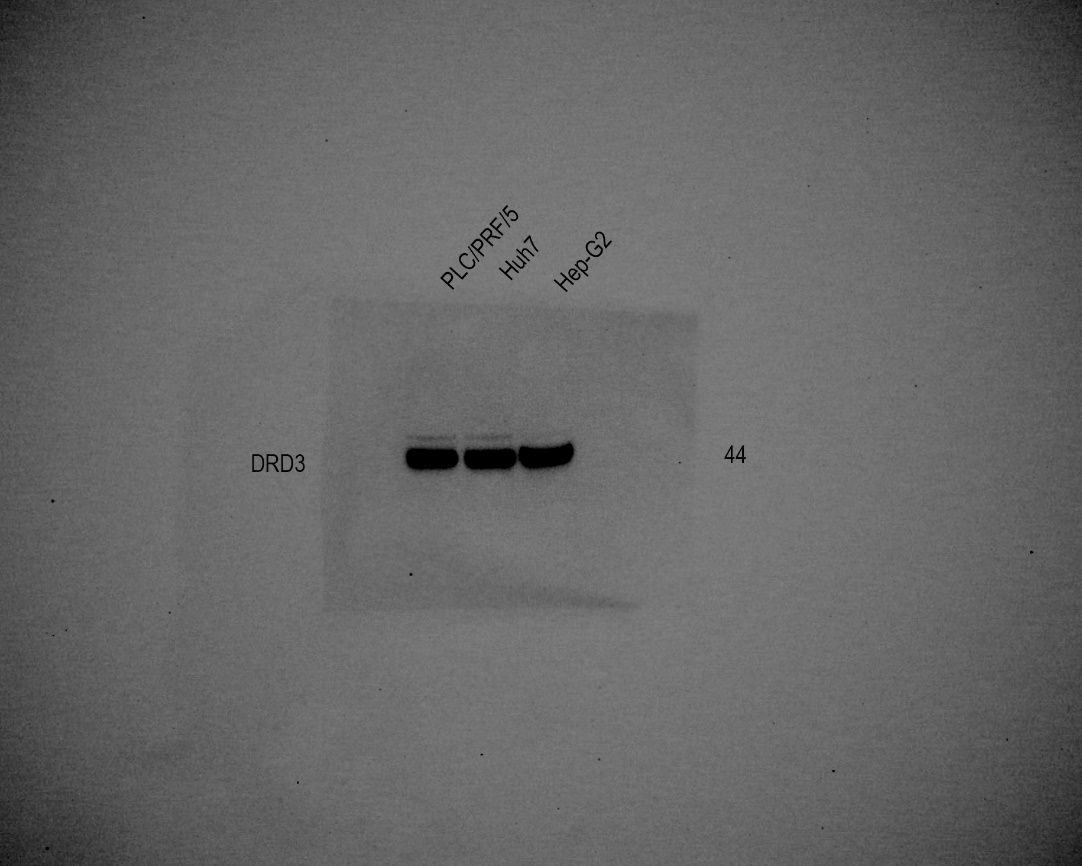


β-tubulin:


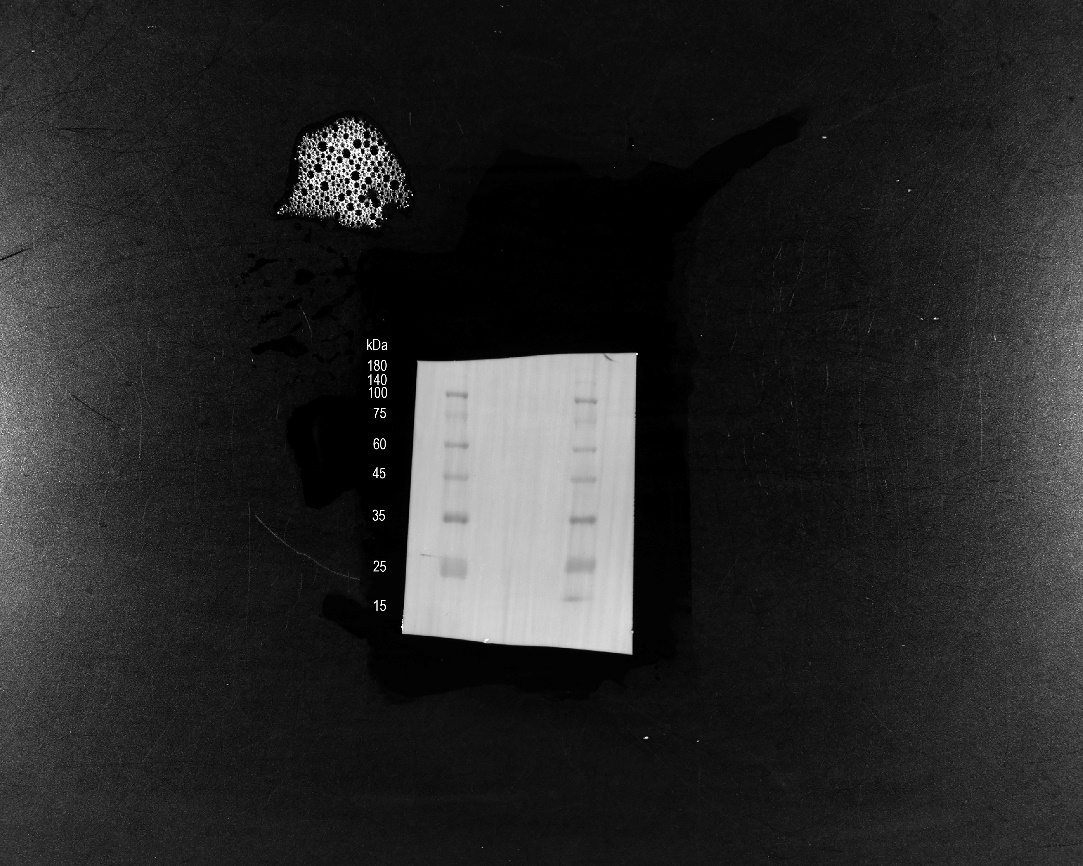

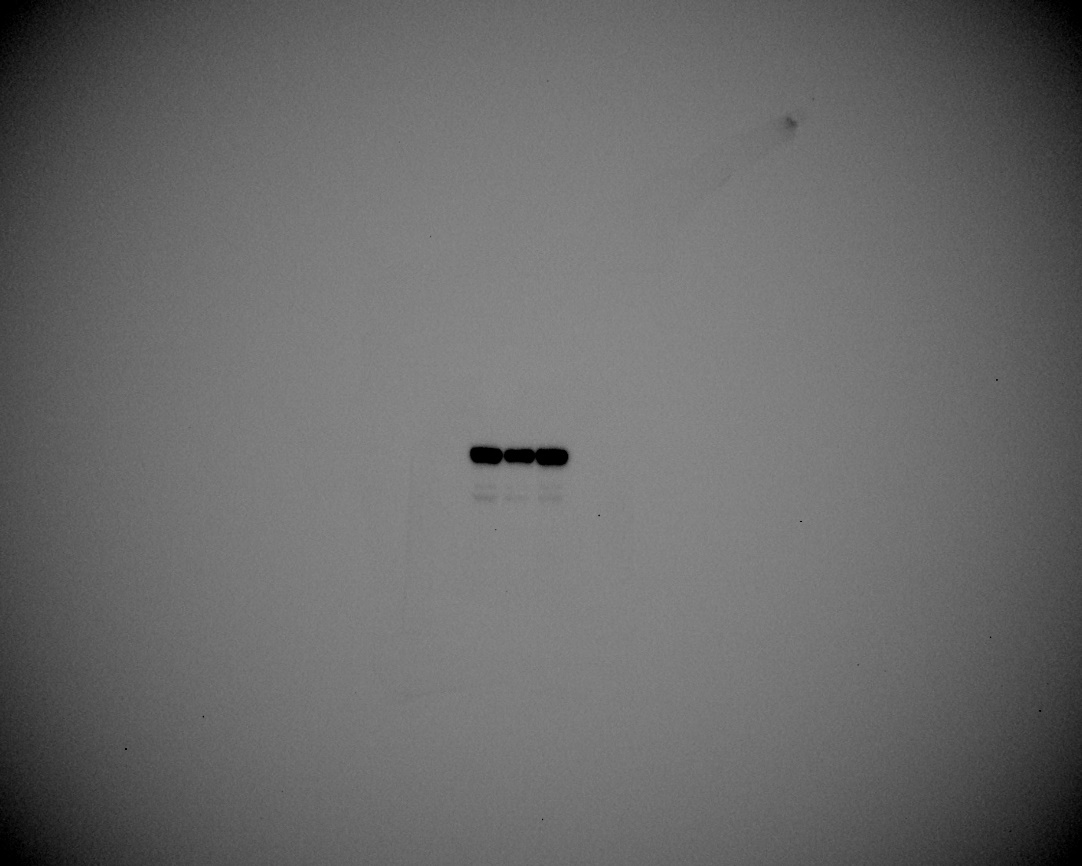

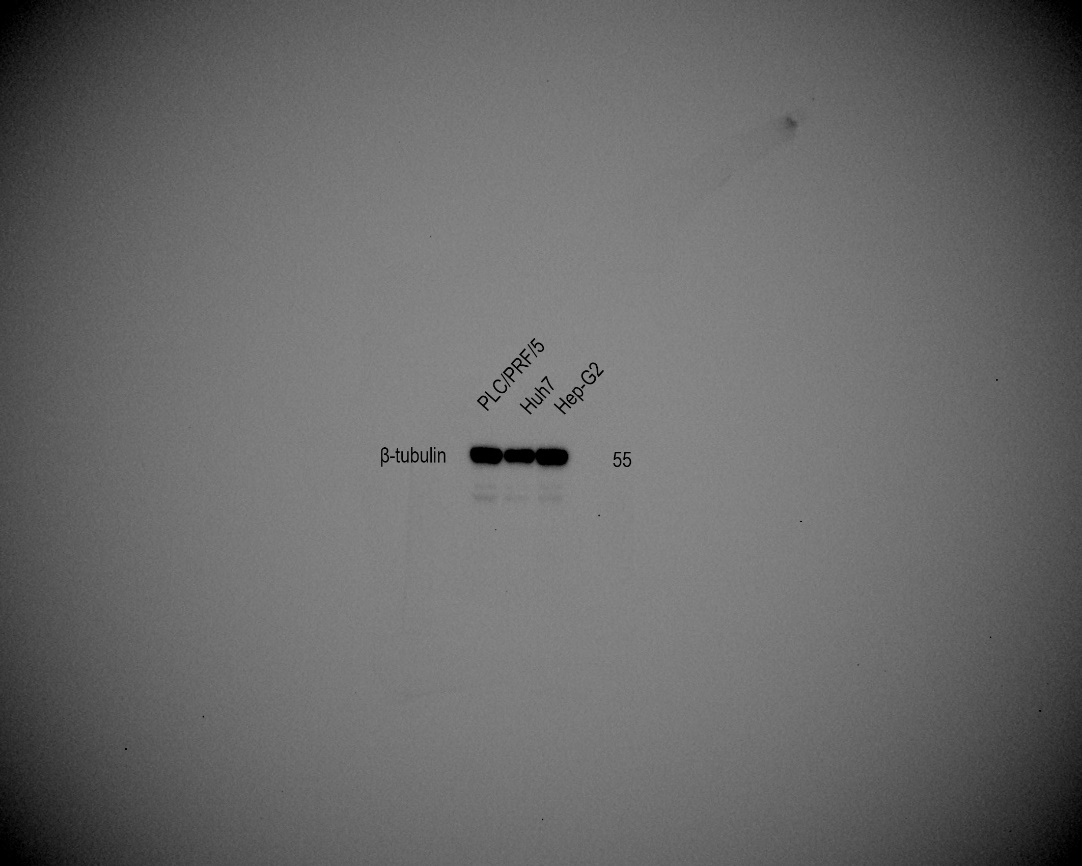


ERK:


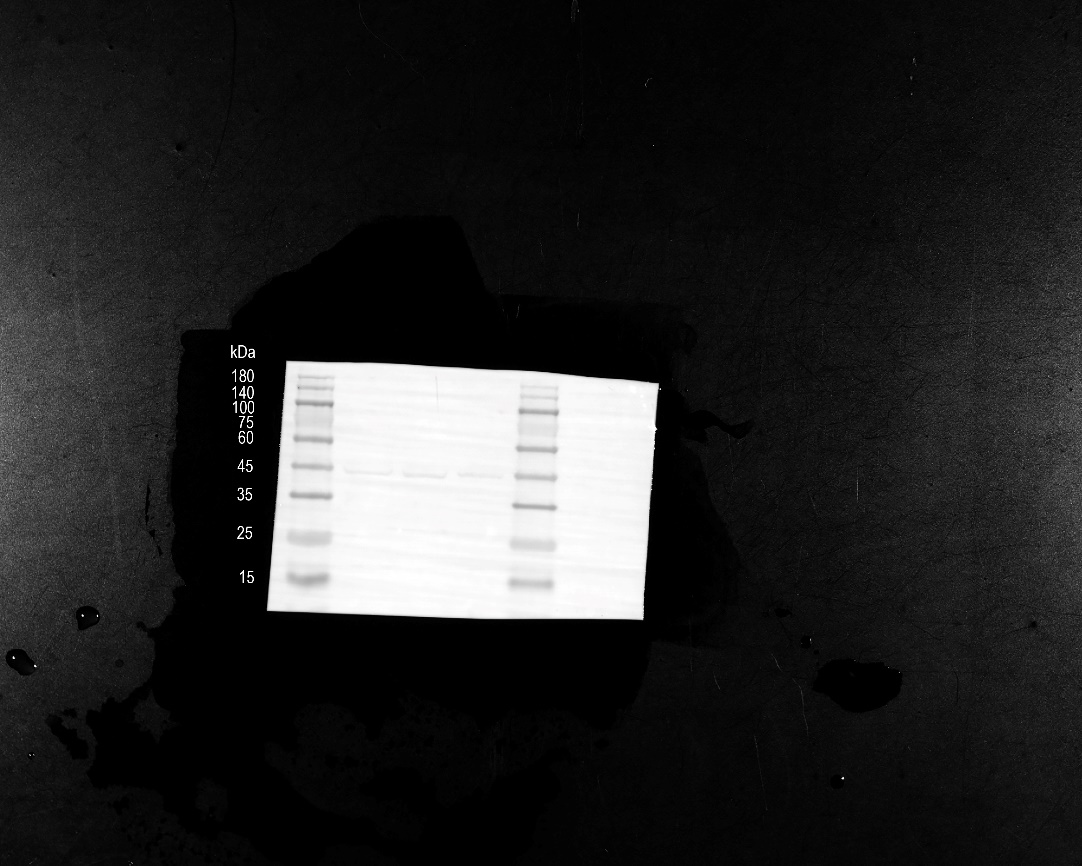


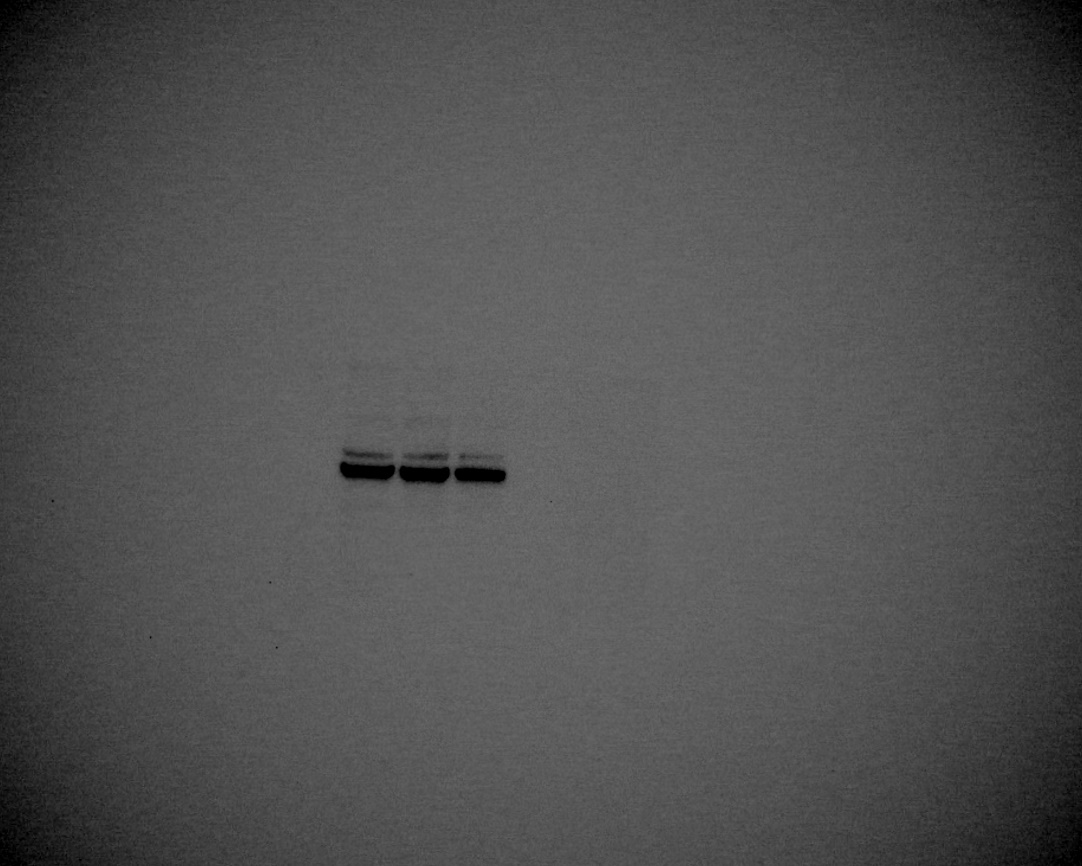

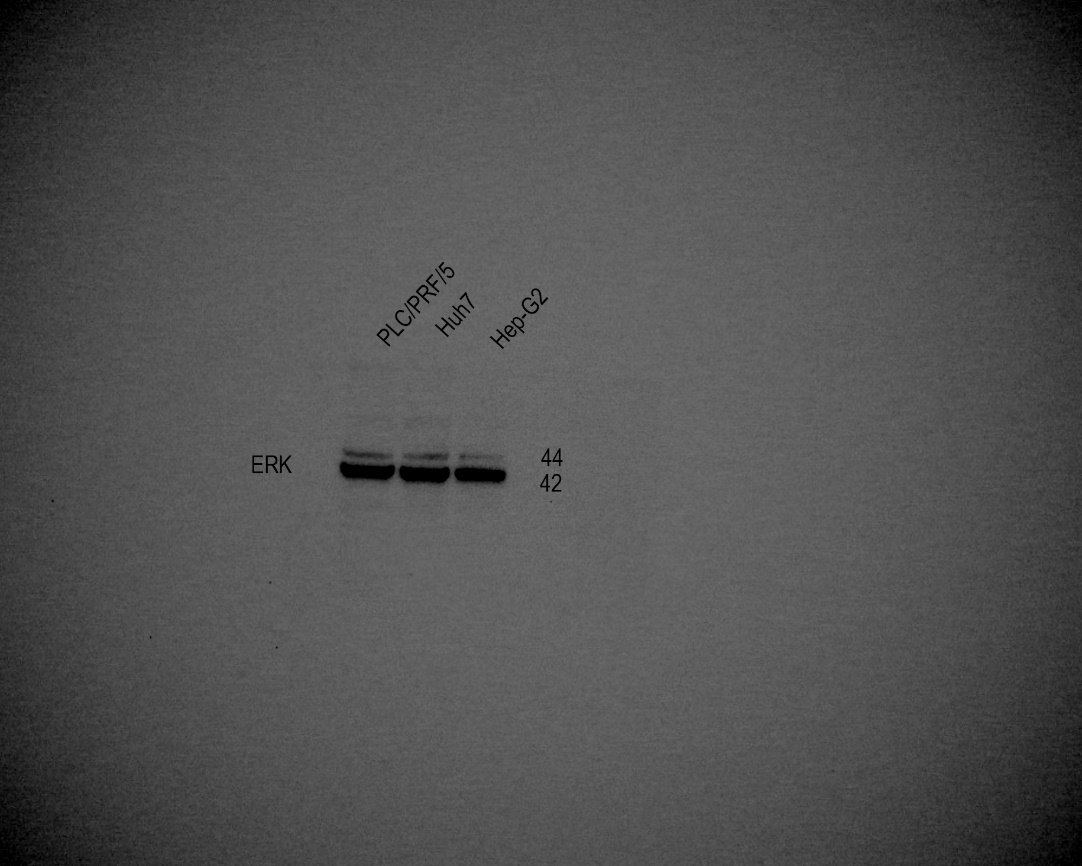


p-ERK:


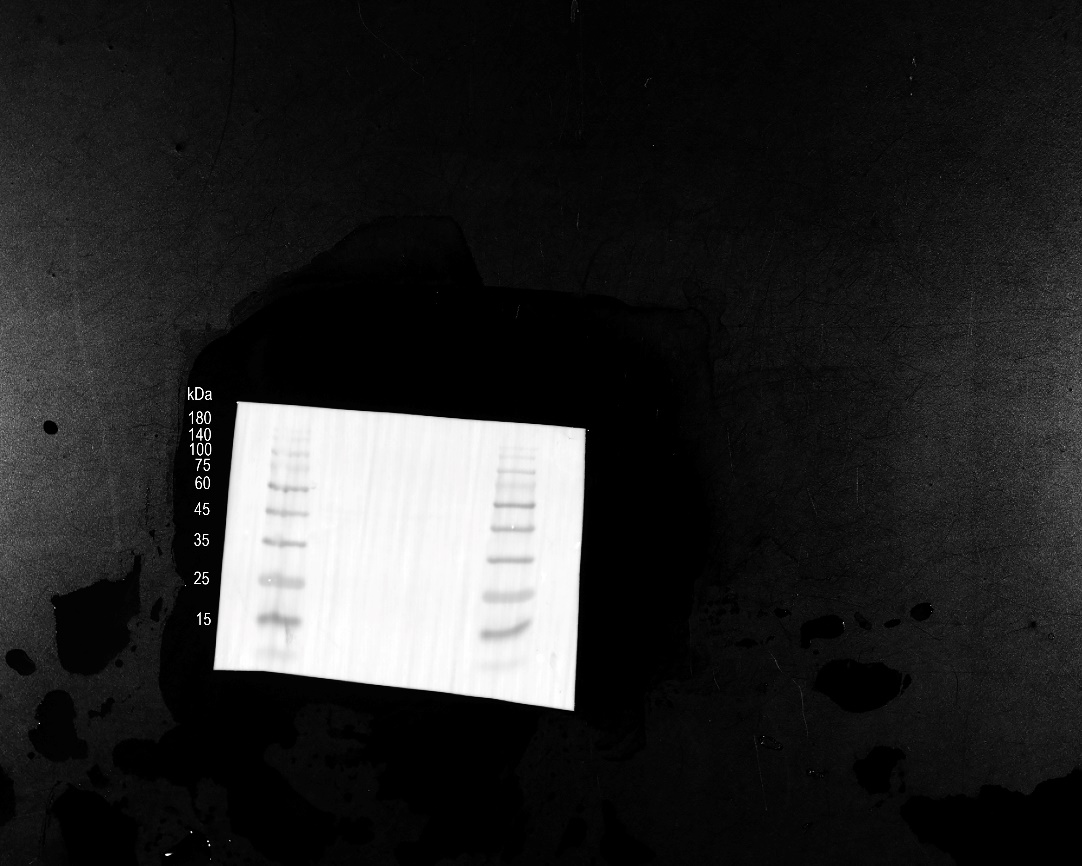

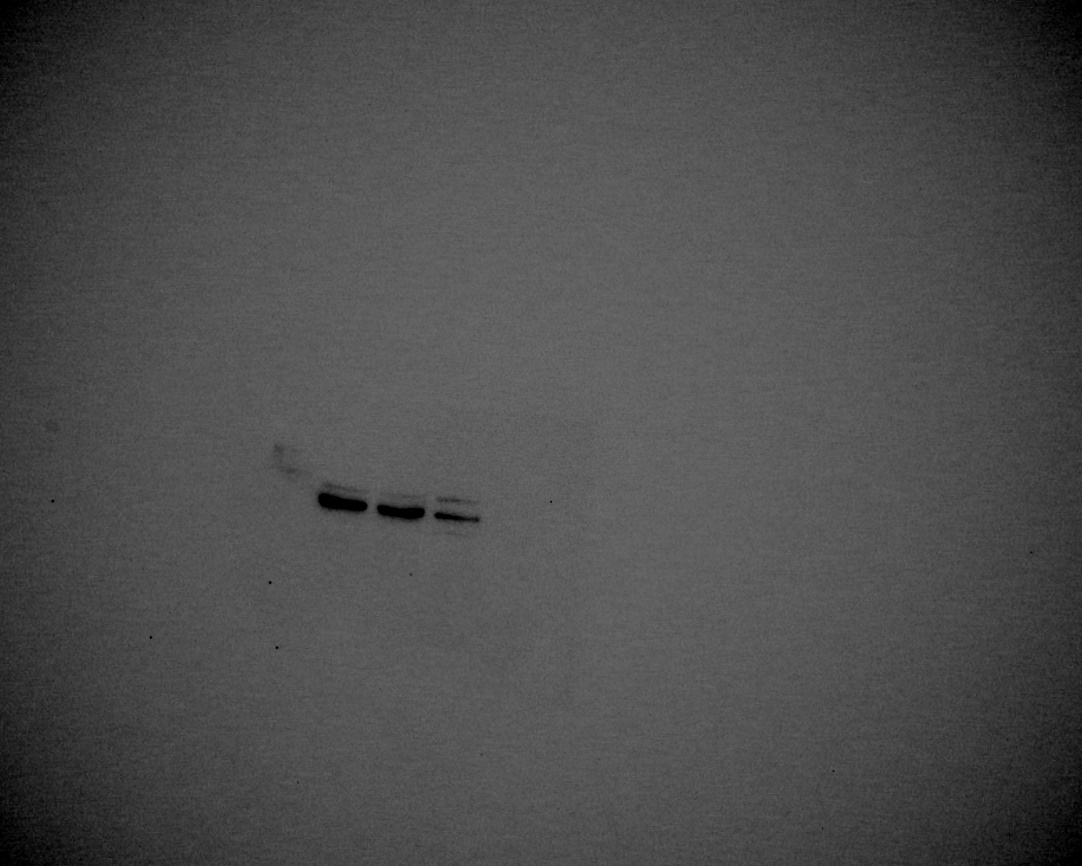


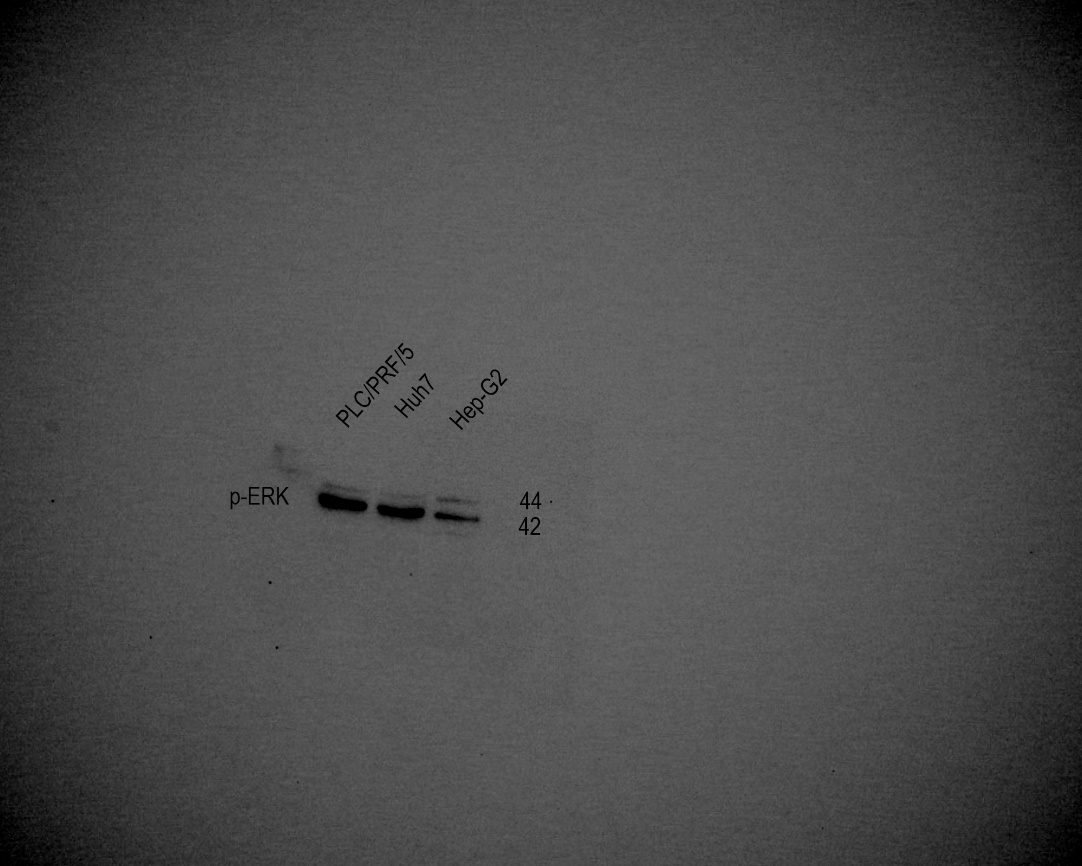


CREB:


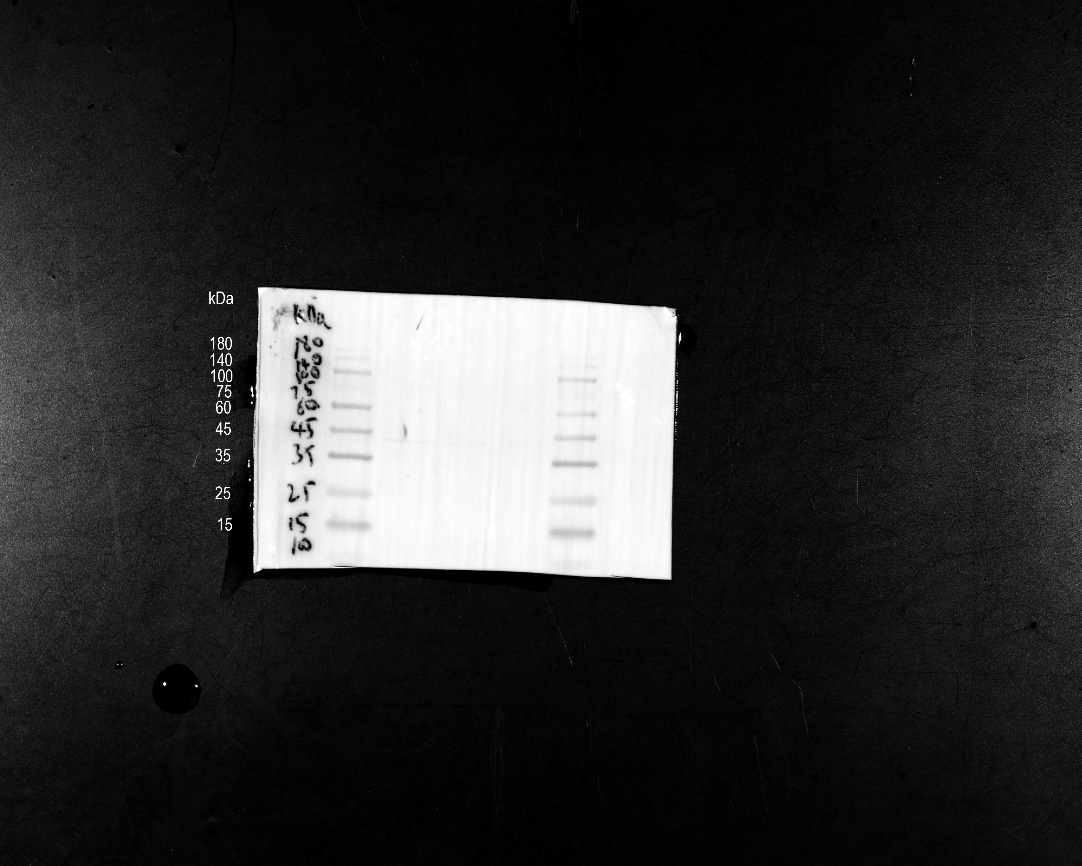

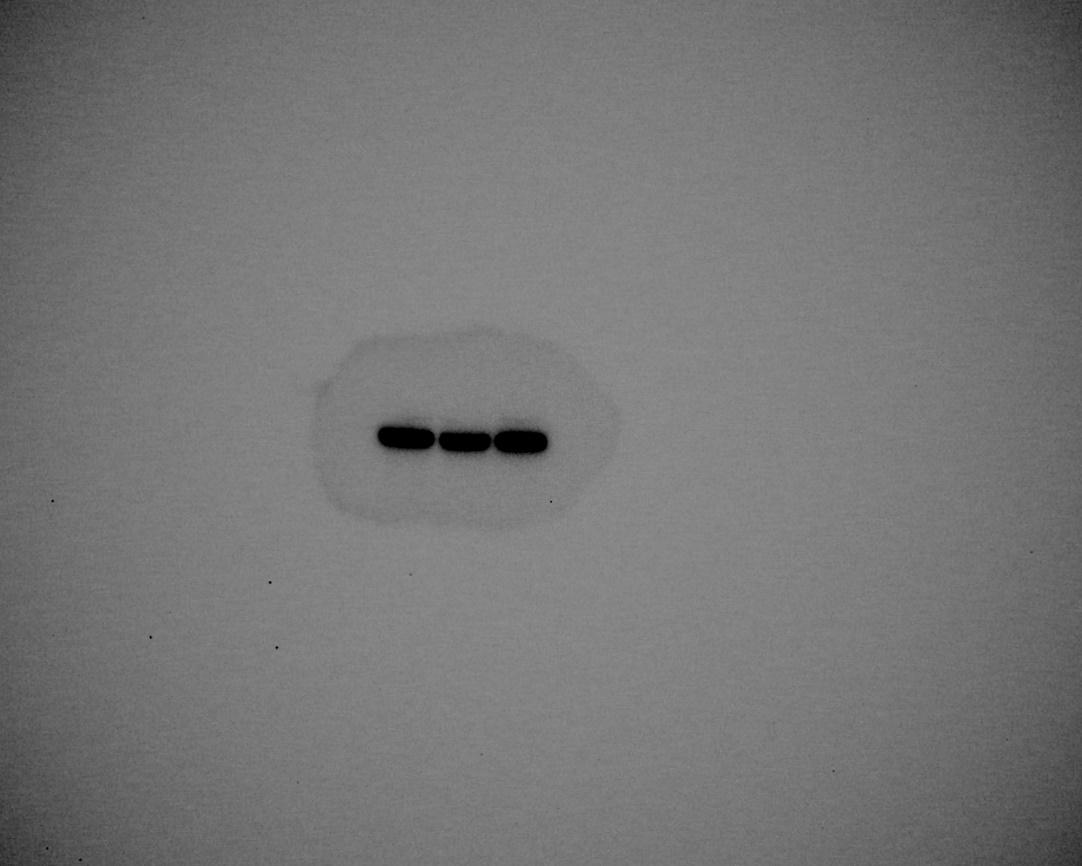

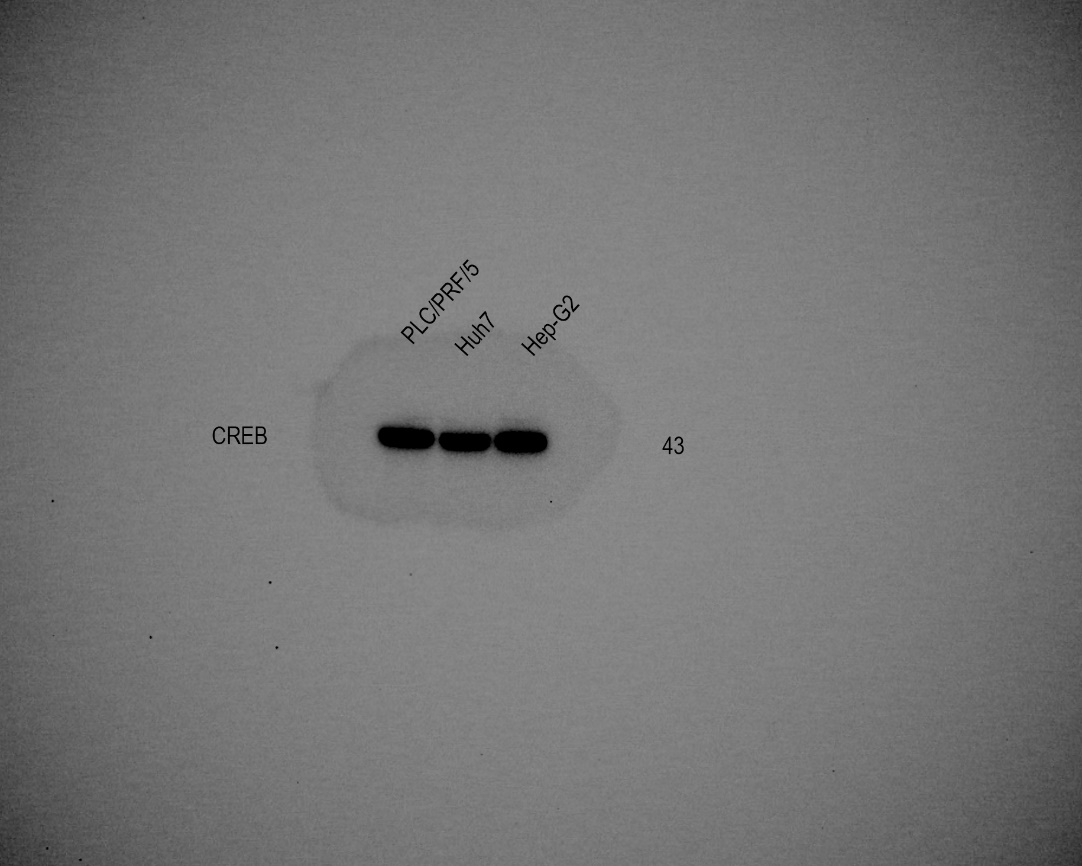


p-CREB:


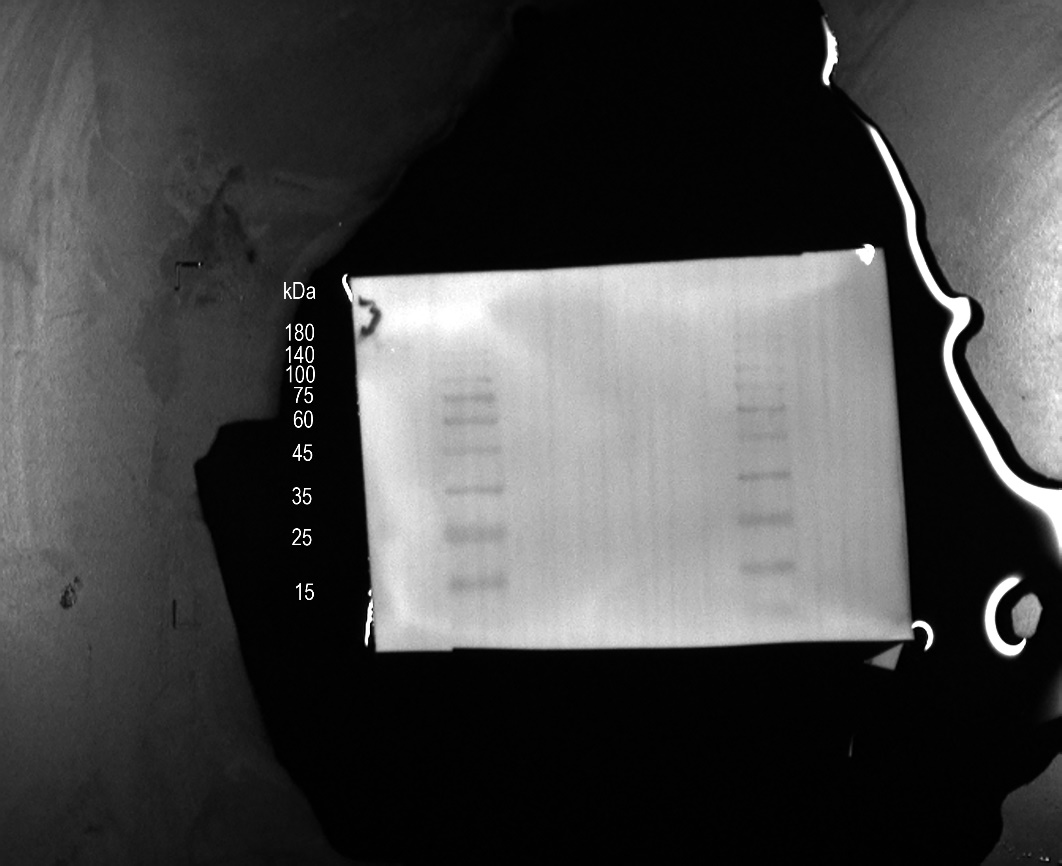



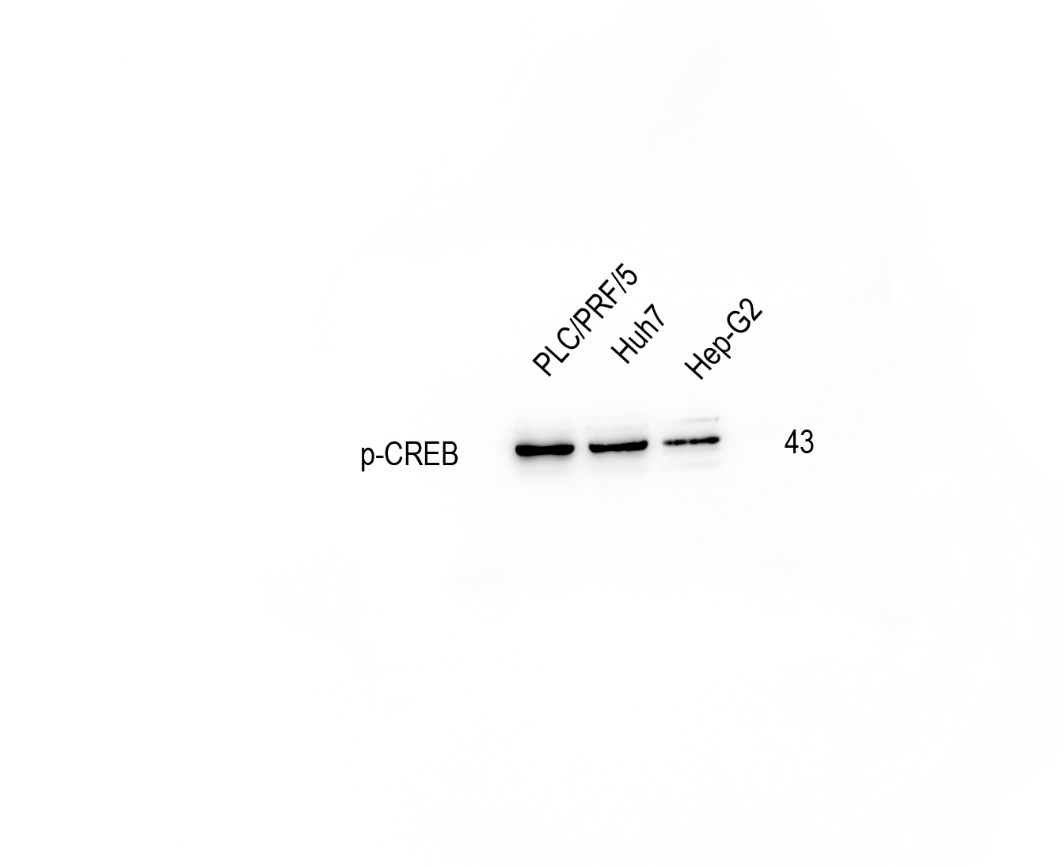

Supplement: Supplementary file 4 — Additional file 4. [file 12885_2022_10368_MOESM4_ESM.docx]
